# Global loss of promoter–enhancer connectivity and rebalancing of gene expression during early colorectal cancer carcinogenesis

**DOI:** 10.1038/s43018-024-00823-z

**Published:** 2024-10-30

**Authors:** Yizhou Zhu, Hayan Lee, Shannon White, Annika K. Weimer, Emma Monte, Aaron Horning, Stephanie A. Nevins, Edward D. Esplin, Kristina Paul, Gat Krieger, Zohar Shipony, Roxanne Chiu, Rozelle Laquindanum, Thomas V. Karathanos, Melissa W. Y. Chua, Meredith Mills, Uri Ladabaum, Teri Longacre, Jeanne Shen, Ariel Jaimovich, Doron Lipson, Anshul Kundaje, William J. Greenleaf, Christina Curtis, James M. Ford, Michael P. Snyder

**Affiliations:** 1 Department of Genetics, Stanford School of Medicine, Stanford, CA USA; 2 Ultima Genomics, Newark, CA USA; 3 Department of Medicine, Stanford School of Medicine, Stanford, CA USA; 4 Department of Pathology, Stanford School of Medicine, Stanford, CA USA; 5 Department of Computer Science, Stanford University, Stanford, CA USA; 6 Chan Zuckerberg Biohub, San Francisco, CA USA

**Keywords:** Chromatin structure, Cancer genomics, Genome-wide analysis of gene expression, Dynamic networks, Cancer

## Abstract

Although three-dimensional (3D) genome architecture is crucial for gene regulation, its role in disease remains elusive. We traced the evolution and malignant transformation of colorectal cancer (CRC) by generating high-resolution chromatin conformation maps of 33 colon samples spanning different stages of early neoplastic growth in persons with familial adenomatous polyposis (FAP). Our analysis revealed a substantial progressive loss of genome-wide cis-regulatory connectivity at early malignancy stages, correlating with nonlinear gene regulation effects. Genes with high promoter–enhancer (P–E) connectivity in unaffected mucosa were not linked to elevated baseline expression but tended to be upregulated in advanced stages. Inhibiting highly connected promoters preferentially represses gene expression in CRC cells compared to normal colonic epithelial cells. Our results suggest a two-phase model whereby neoplastic transformation reduces P–E connectivity from a redundant state to a rate-limiting one for transcriptional levels, highlighting the intricate interplay between 3D genome architecture and gene regulation during early CRC progression.

## Abstract (web summary)

Snyder and colleagues show global loss of promoter–enhancer connectivity during early colorectal carcinogenesis and the dependency of gene dysregulation during this process on the baseline of promoter–enhancer interaction in normal colonic epithelium.

## Main

The technological advent of three-dimensional (3D) chromosome organization mapping has revealed important insights into genome folding^1–3^. Multilayered structures maintained by molecular contacts, insulators and aggregative domains together compact the 2-m DNA into nonrandom spatial configurations in the nucleus^2,4^. However, the functional implications of such spatial organization on fundamental biological processes remain largely elusive.

A key discovery in genome topology is the formation of topologically associating domains (TADs)^5,6^, high-order structures that partition the genome into contiguous regions through a proposed loop extrusion mechanism^7,8^. While genetic mutations affecting TAD structures have been linked to oncogenic gene dysregulations in specific cases^9–11^, the exact role of TAD organization in transcription regulation remains an open question. Recent studies have shown a surprisingly moderate transcriptional response to the manipulation of boundary elements such as CTCF and cohesin components^12,13^. Furthermore, computational approaches have suggested a lack of coexpression between genes residing in the same TAD^14,15^. These findings imply that gene regulation is often not particularly constrained by large submegabase folding domains but rather depends on a finer layer of regulatory architecture at the sub-TAD level.

Recent advancements in chromatin conformation capture technologies, using micrococcal nuclease^16,17^ or a combination of restriction enzymes^18,19^, have improved mapping resolution and enhanced the detection of sub-TAD structures. These methodologies have uncovered prevalent distal interaction activities, including architectural stripes and insulation activities associated with active *cis*-regulatory elements (CREs). However, the functional implications of these structures remain largely uncharacterized. Moreover, studies of transcriptional kinetics based on imaging^20,21^ and multiomics sequencing^22,23^ have revealed disjoined changes between the spatial proximity of regulatory elements and transcriptional activation events. These observations highlight the intricate role of physical connectivity in gene regulation.

Colorectal cancer (CRC) represents a major global health burden and is the second leading cause of cancer death in the United States^24^. Over 80% of colorectal carcinomas are initiated by loss-of-function mutations of adenomatous polyposis coli (APC), a key component in the cytosolic complex that targets β-catenin for destruction and suppresses Wnt signaling^25,26^. Persons with familial adenomatous polyposis (FAP) carry germline *APC* mutations and develop tens to thousands of precancerous polyps at different stages and sizes, as well as occasional adenocarcinomas; these polyps are believed to represent early stages of CRC^27,28^. Thus, the study of polyps at different stages of development in persons with FAP provides a valuable model for studying the cascades of epimutations and gene dysregulations during early oncogenesis.

In the context of the Human Tumor Atlas Network (HTAN)^29^, we profiled genome-wide chromatin conformation at up to 100-bp resolution in 33 colon samples from persons with FAP and CRC representing different stages of CRC progression. By integrating these data with transcriptome and epigenome profiling, we investigated the relationship between fine-gauge chromatin structures organized by active regulatory elements and gene dysregulation associated with polyp malignancy. Our analysis revealed a progressive loss of *cis*-regulatory connectivity from mucosa to polyps to adenocarcinoma, corresponding with dysregulated gene expression in a nonlinear fashion. The initial connectivity levels before oncogenic progression may be a key factor in this process. We propose that the remodeling of the promoter–enhancer (P–E) connectome is not only indicative of alterations of gene expression but also reflects shifts in the transcriptional response to epigenetic alterations in polyps and adenocarcinoma. Our rich dataset provides a valuable resource for unraveling the chromatin architectural basis of early CRC development.

## Results

### Fine mapping of regulatory element interactions using mHi-C

**Fig. 1:**
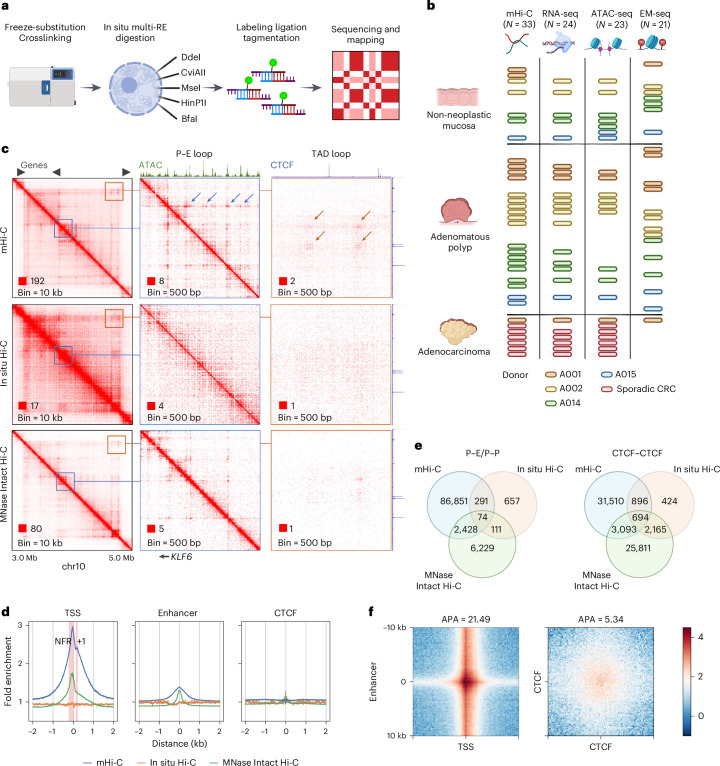
mHi-C reveals interactions associated with active CREs. **a**, Schematic representation of the mHi-C workflow. **b**, Summary of colon tissue samples analyzed by multiomics assays. Bar colors represent different donors. Each row corresponds to a unique donor with sporadic CRC. The number of biospecimens examined by each assay: *n* = 33 for mHi-C, *n* = 24 for RNA-seq, *n* = 23 for ATAC-seq and *n* = 21 for EM-seq. **c**, Comparison of contact matrices generated from mHi-C (combined colon tissues) with in situ Hi-C (HCT116) and MNase-digested Intact Hi-C (HCT116) at various resolutions. Blue arrows highlight interaction dots formed between the *KLF6* promoter and adjacent enhancers. Orange arrows show structural loops at TAD boundaries. **d**, Venn diagrams illustrating the overlap of interaction loops identified by the three methods. **e**, Average fold enrichment (FE) of distal interactions at TSSs, active enhancers and CTCF-binding sites in mucosa samples. Red intervals indicate the nucleosome-free region (NFR) and the +1 nucleosome regions upstream and downstream of the TSS, respectively. **f**, APA of loops between promoters and active enhancers (*n* = 9,174) and between CTCFs (*n* = 30,208). Source data

**Extended Data Fig. 1:**
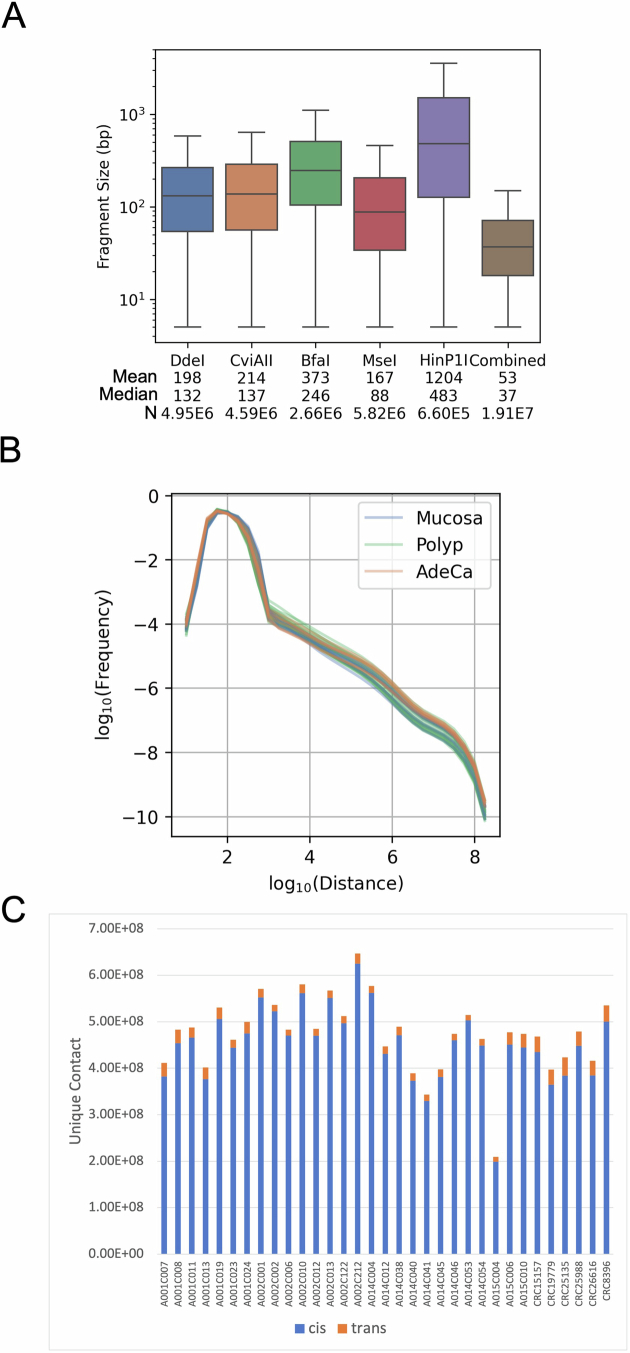
Summary statistics of mHi-C. (**a**) Fragment size distribution of human genome (hg38) digested by the indicated restriction enzymes and their combination. Restriction site count: DdeI=4.95E6, CviAII=4.59E6, BfaI=2.66E6, MseI=5.82E6, HinP1I = 6.60E5, combined=1.91E7. (**b**) Frequency of intrachromosomal interactions by genomic distance for various sample types. (**c**) Count of unique interaction contacts identified across samples. Source data

To examine the chromatin architecture associated with regulatory elements in clinical tissue samples, we developed multidigested Hi-C (mHi-C), a protocol derived from in situ Hi-C^30^ that uses five 4-bp-cutter restriction enzymes and moderated detergent conditions to achieve ultrafine mapping (mean fragment size = 52 bp) of the distal chromatin interactions^18,31^ (Fig. 1a, Extended Data Fig. 1a and Methods). We generated mHi-C data for 33 frozen colon tissue samples at different stages of neogenesis (Fig. 1b and Supplementary Table 1), comprising seven non-neoplastic mucosa, 19 dysplastic polyps and one adenocarcinoma from four persons with FAP, as well as six additional adenocarcinoma samples from non-FAP individuals who developed sporadic CRC. A total of 1.59 billion unique intrachromosomal long-range (≥1 kb) interaction contacts were mapped (Extended Data Fig. 1b,c).

**Extended Data Fig. 2:**
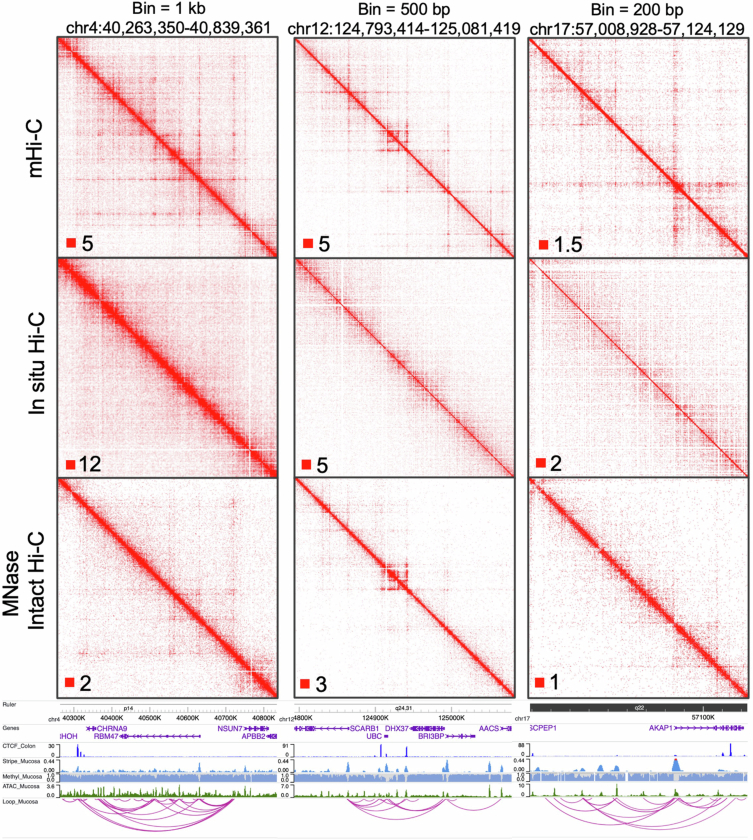
Comparison of mHi-C with in situ Hi-C and MNase-digested Intact Hi-C at diverse resolutions. Upper panel shows contact heatmaps of three genomic loci obtained by these methods at indicated bin resolution. Annotated genes, open chromatin regions, CTCF binding sites, as well as architectural stripes and loops called from mHi-C are indicated in the lower panel.

**Extended Data Fig. 3:**
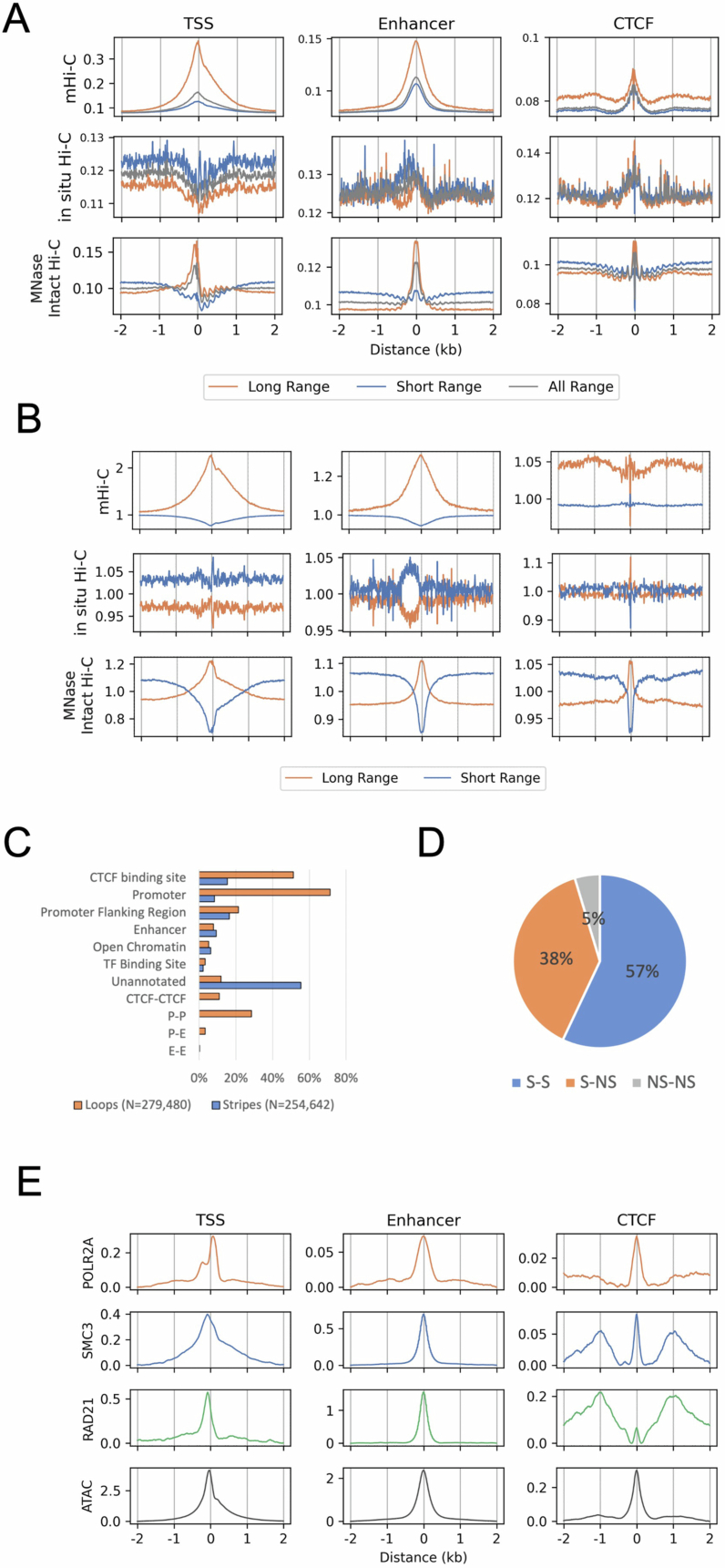
mHi-C delineates interaction features at active CREs. (**a**) Aggregated read intensity of long- (>1.5 kb) and short-range (<1.0 kb) interactions before and (**b**) after normalizing against total coverage (All range) at distinct CRE categories. (**c**) Classification and annotation of identified stripes and loops in colon samples by regulatory element types. (**d**) Proportion of loops composed of two stripe anchors (S-S), between a stripe anchor and a non-stripe anchor (S-NS), and two non-stripe anchors (NS-NS). (**e**) Aggregated ChIP-seq signals for Pol II, SMC3, Rad21, and ATAC seq fold enrichment at various CRE types in colon samples, sourced from ENCODE data. Source data

Similar to micrococcal nuclease (MNase)-digested Intact Hi-C in the ENCODE consortium^32^, mHi-C robustly revealed fine-gauge structures at subkilobase resolution (200 bp–1 kb). This included dot interactions, indicative of looping of two fixed anchors, and architectural stripes, indicative of dynamic looping between a fixed anchor and the sliding intervening neighboring regions (Fig. 1c and Extended Data Fig. 2)^7,8^. Previous studies have correlated these structures with interaction hotspots identified by high-depth 4C assays, enriched at regulatory elements^18,33,34^. To annotate the interacting regions, we mapped open chromatin regions in 23 matched samples using an assay for transposase-accessible chromatin with sequencing (ATAC-seq)^35^ (Fig. 1b) and annotated the regulatory elements using the Ensembl regulatory build^36^. Notably, both MNase-digested Intact Hi-C and mHi-C but not in situ Hi-C revealed enriched interactions at promoters and enhancers (Extended Data Fig. 3a). This enrichment was specific to long-range contacts, which persisted after normalizing against short-range or total read coverage (Fig. 1d and Extended Data Fig. 3b), indicating that the observed stripe signals are not artifacts of differential contact mappability because of the high accessibility of these regions.

Using the HICCUPs algorithm^37^, we identified 279,480 loop interactions, including 91,706 promoter–promoter (P–P) or P–E contacts (Extended Data Fig. 3c). Compared to chromatin conformation profiles obtained from MNase-digested Intact Hi-C and in situ Hi-C, mHi-C identified approximately 10-fold and 100-fold more P–E and P–P interactions, but only 1.5-fold and 8.5-fold more loops between CTCF-binding sites, respectively (Fig. 1e). This suggests that mHi-C specifically improves the detectability of contacts among active regulatory elements. Meanwhile, using a peak calling algorithm based on MACS2 (refs. ^18,38^), we identified 254,642 stripes in all samples (Methods). Of the 279,480 loops, 266,419 (95%) overlapped with stripes (Extended Data Fig. 3d), indicating that the majority of loop contacts are formed along with stripe extension.

**Fig. 2:**
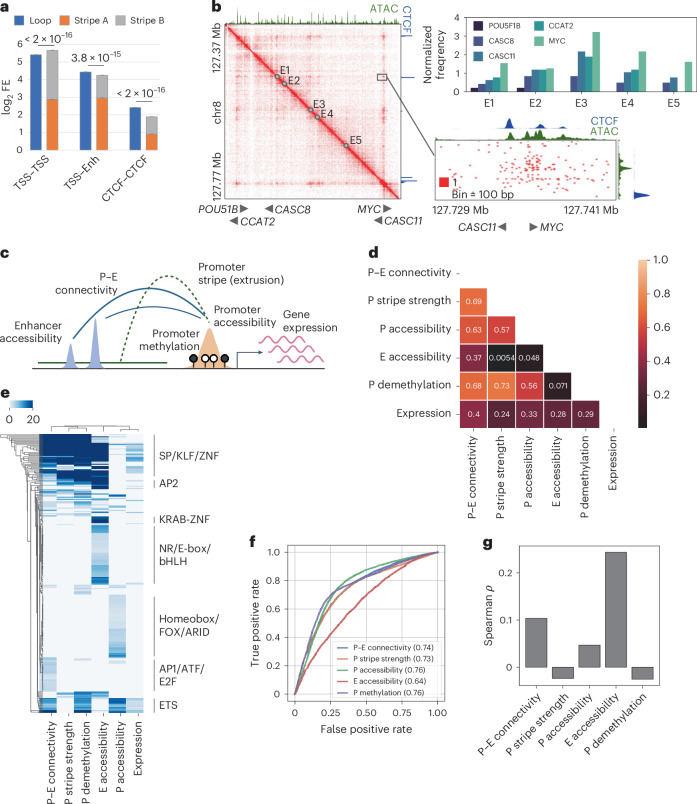
Correlation between promoter stripe formation and P–E connectivity. **a**, Comparison of mean loop strengths with the logarithmic sum of mean stripe strengths at the anchors for loops formed between different regulatory elements. Error bars represent confidence intervals. Statistical significance was assessed using the Wilcoxon signed-rank test. **b**, Contact heat map (combined colon tissues) of the *MYC* upstream cancer risk locus. Top right, contact frequencies of resident genes with the five putative enhancers exhibiting the highest distal interaction activity. Bottom right, a detailed view of contact distribution between enhancer E1 and the *MYC* and *CASC11* genes. **c**, Schematic representation of the integration of conformational, epigenetic and transcriptional features for downstream analysis. **d**, Spearman correlation matrix of average feature strengths in mucosa samples for all examined coding genes (*n* = 14,692). **e**, Hierarchical clustering of genes exhibiting top 10% intensity for any of the six examined features, based on the degree of motif enrichment (adjusted −log_10_(*P* value)) on their promoters. **f**, ROC analysis for predicting actively expressed genes (TPM > 0.5, *n* = 10,663) using various structural and epigenetic features. Numbers indicate the AUC scores. **g**, Spearman correlation of the expression levels of actively expressed genes with the examined features. Source data

**Extended Data Fig. 4:**
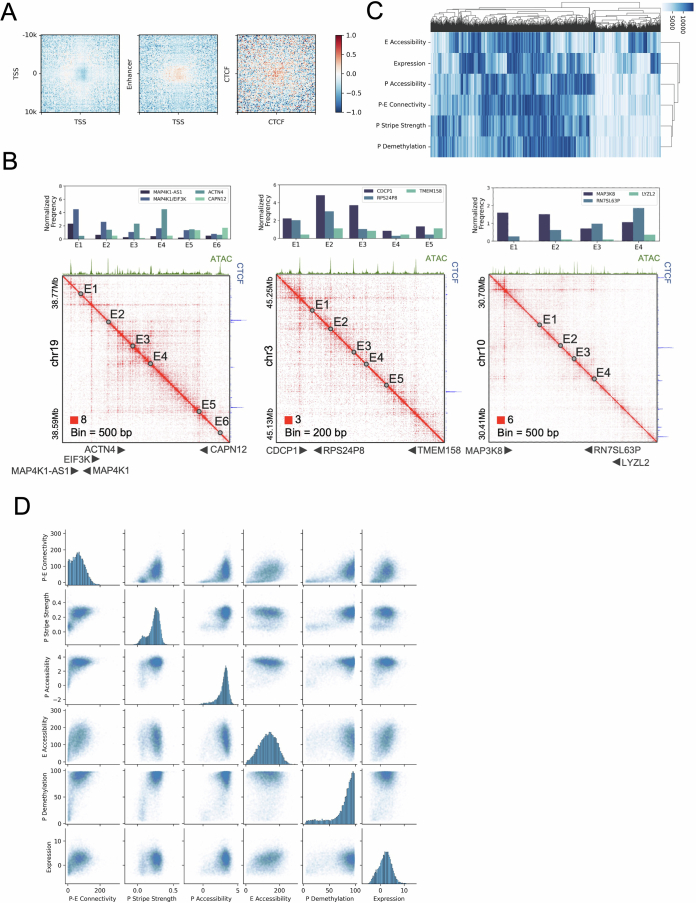
Analysis of the interplay between structural features and epigenetic markers. (**a**) Heatmaps displaying loop signal intensities as referred to in Fig. 2a, adjusted for the effects of stripe strengths at the loop anchors. (**b**) Contact heatmap at example loci where gene promoters lacking CTCF binding display gene-specific P-E interactions. (**c**) Hierarchical clustering of genes based on their rankings for various structural and epigenetic features in mucosa samples. Intensity of color corresponds to the strength of the features. (**d**) Comparative scatter plots illustrating the relationships between different structural and epigenetic features across the examined dataset. Source data

At 100-bp resolution, we observed a pronounced difference between P–E and CTCF–CTCF interactions. Whereas contacts between promoters and enhancers are formed at open chromatin regions, CTCF interactions do not strictly overlap with CTCF-binding sites. Instead, they spread widely within a multikilobase flank region (Fig. 1f). These patterns are consistent with occupancy of the cohesin components at the open chromatin of active regulatory elements, as opposed to being retained at a broader region around CTCF sites (Extended Data Fig. 3e). Furthermore, we found that the loop signals of P–E interactions were comparable with the sum of the anchors’ stripe strengths, whereas the loop strengths of CTCF interactions significantly exceeded their architectural stripe strengths (Fig. 2a and Extended Data Fig. 4a). This difference suggests that, unlike CTCF boundaries, which can maintain stable loop structures^39^, P–E interactions are dynamically maintained while the intervening anchors interact frequently with each other’s neighborhoods.

### Promoter stripes shape gene-specific P–E connectivity

We observed that P–E interactions are asymmetrically contributed by the relatively stronger stripe-forming activity of the promoters and weaker activity of the enhancers (Figs. 1f and 2a). This asymmetry underscores the dominant role of promoters in shaping the P–E connectivity. To validate this hypothesis, we conducted a case study of the *MYC* upstream locus, which is a known risk hotspot for multiple cancer types^40^ that resides near five coding genes (*POU51B*, *CCAT2*, *CASC8*, *CASC11* and *MYC*) and multiple enhancers. Our results demonstrated that *MYC*, which exhibited the highest stripe strength, consistently interacted with all enhancers with the highest frequency among all five genes, despite other genes being located closer to these enhancers (Fig. 2b). Remarkably, the enhancers tended to bypass *CASC11*, a gene located only 1 kb upstream of *MYC* with substantial promoter CTCF binding, and instead favored robust interactions with the *MYC* promoter, which showed lower CTCF affinity. We extended our examination to additional loci and consistently found that gene-specific promoter stripe activities led to distinctive interaction profiles for genes sharing the same enhancer context (Extended Data Fig. 4b).

To further investigate the relationship between P–E connectivity and promoter or enhancer activity, we profiled the landscapes of methylome and transcriptome from the matching FAP and CRC samples (Fig. 1b). We quantified the connectivity of all coding genes with their neighboring enhancers within a 200-kb distance. We then correlated this connectivity with the accessibility of the enhancers, the methylation and stripe activity of the gene promoters, assessed by the fold enrichment of the overlapping architectural stripes, in unaffected mucosa (Fig. 2c). The connectivity exhibited a strong correlation with stripe strength (*ρ* = 0.69), demethylation (*ρ* = 0.68) and accessibility (*ρ* = 0.63) of the gene promoter. By contrast, its correlation with enhancer accessibility was much lower (*ρ* = 0.37) (Fig. 2d). Consistently, clustering analysis revealed distinct groups of genes associated with high connectivity but low-to-moderate enhancer accessibility and vice versa, indicating a discrepancy between the availability of enhancers and their connectivity with promoters (Extended Data Fig. 4c). Motif analysis uncovered a strong enrichment of transcription factors (TFs) with G+C-rich motifs on highly interactive promoters, distinguishing them from promoters associated with high accessibility or rich neighboring enhancer contexts (Fig. 2e). Collectively, these results suggest that the degree of P–E connectivity of genes is predominantly correlated with the stripe activity on their promoters, corroborating our observations in the case studies.

Upon observing a substantial divergence between the promoter’s connectivity and the availability of distal regulatory elements, we investigated which factor was more relevant to transcription. We discovered that the overall correlation of connectivity with gene expression surpassed that of enhancer accessibility (Fig. 2d). However, this high correlation predominantly stemmed from the association of transcriptionally inactive genes (transcripts per million (TPM) < 0.5) with low connectivity (Extended Data Fig. 4d). As such, high connectivity serves as a robust indicator of an ‘on’ state for promoters, similar to their accessibility, demethylation and stripe strength (Fig. 2f). Conversely, the expression level of active genes (TPM > 0.5) was poorly correlated with the connectivity (*ρ* = 0.10) but rather explained by the enhancer accessibility (*ρ* = 0.24) (Fig. 2g). This implies that regulation of quantitative expression was dependent on the activity of enhancer context for genes with connectivity passing beyond the ‘on’ threshold.

### Impaired P–E connectivity indicates promoter instability

**Fig. 3:**
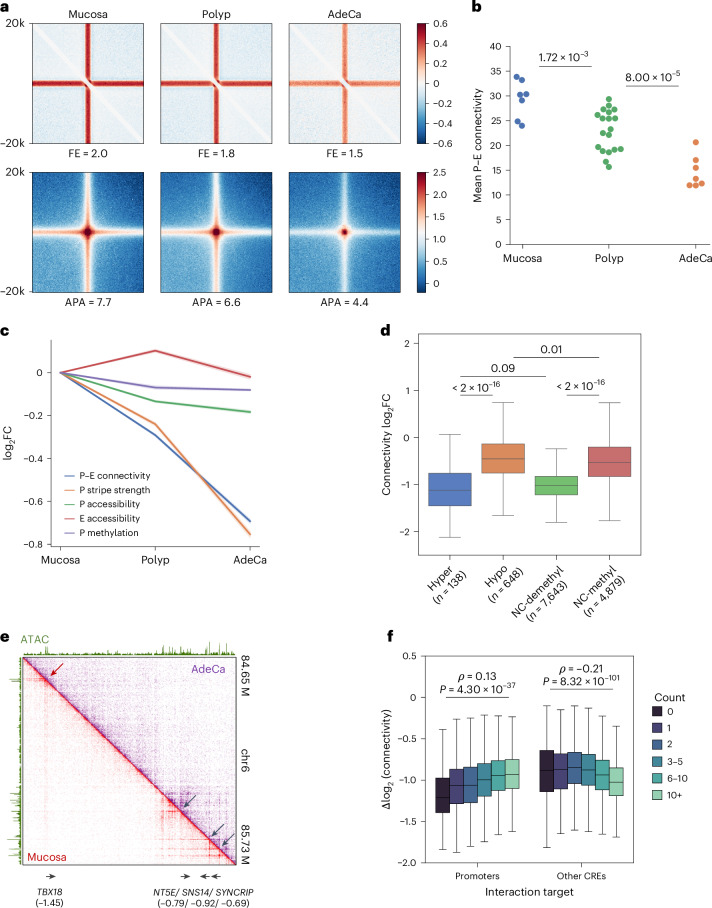
Loss of distal connectivity in polyps and adenocarcinoma. **a**, APA of all stripe (top) and loop (bottom) anchors, with FE of signals indicated below the panels. **b**, Mean P–E connectivity for coding genes in mucosa, polyp and adenocarcinoma samples. The sample sizes of each stage: *n* = 7 for mucosa, *n* = 19 for polyp and *n* = 7 for adenocarcinoma. The *P* values for stage comparisons were determined by the Mann–Whitney *U*-test. Mucosa versus polyp, *P* = 1.72 × 10^−3^; polyp versus adenocarcinoma, *P* = 8.00 × 10^−5^. **c**, Average log_2_ fold changes of structural and epigenetic features in polyps and adenocarcinoma, with confidence intervals represented by shaded areas. **d**, Changes in connectivity between mucosa and adenocarcinoma for genes categorized by hypermethylation, hypomethylation or no change (NC, <5% difference) in methylation status. Groups with demethylated (<25%) and methylated (>40%) promoters are compared. The number of promoters in each category: *n* = 138 for hypermethylated, *n* = 648 for hypomethylated, *n* = 7,643 for NC-demethylated and *n* = 4,879 for NC-methylated. **e**, Comparison of contact heat maps for a representative locus in mucosa and adenocarcinoma samples, with log_2_ connectivity changes for gene promoters indicated below. **f**, Comparison of connectivity changes for genes (*n* = 9,901) interacting with varying numbers of promoters and other CREs, including Spearman correlation coefficients and *P* values from the Mann–Whitney *U*-test. Source data

**Extended Data Fig. 5:**
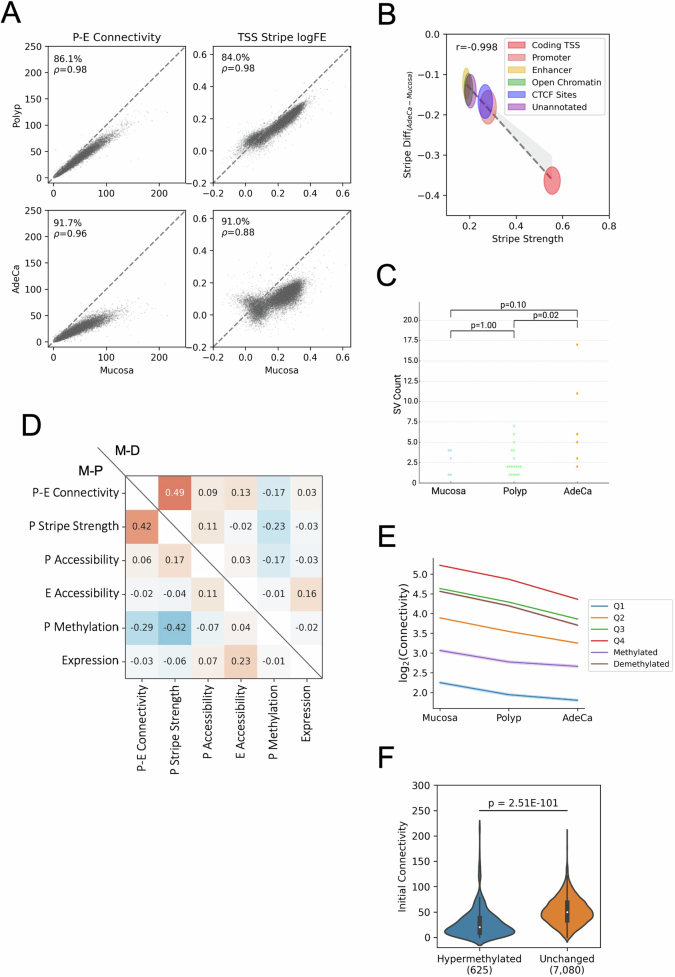
Dynamics of P-E connectivity through colorectal cancer (CRC) progression. (**a**) Scatter plots depicting the comparative analysis of P-E connectivity and TSS stripe strengths across different CRC stages. The percentage of genes with reduced connectivity (y < x) during progression is indicated for each stage comparison. (**b**) Correlation between initial stripe strength in mucosa samples and the extent of stripe reduction in adenocarcinoma samples. Each ellipse’s center and radius represent the mean and standard deviation, respectively, for stripes associated with the specified regulatory elements. The dotted line shows the linear regression across the centers of the ellipses. (**c**) Distribution of SV counts in samples. Significance p values of count differences stages from Mann–Whitney U test are indicated. (**d**) Spearman correlation matrix detailing the changes (log2 fold change, FC) of structural and epigenetic features between polyps (M-P) and adenocarcinoma (M-D) relative to unaffected mucosa. (**e**) Average log2 fold change in P-E connectivity for polyps and adenocarcinoma, categorized by promoter methylation status: quantiles (Q1-Q4), demethylated, and methylated, excluding those with minimal hypo- or hyper-methylation. Confidence intervals are depicted as shaded areas behind each line. (**f**) Distribution of P-E connectivity in mucosa samples for gene promoters that become hypermethylated (N = 625) or remain unchanged (N = 7,080) in adenocarcinoma. The significance of differences (p = 2.51E-101) is tested using the Mann-Whitney U test. Source data

During the development of CRC malignancy, we observed that more than 80% of identified stripes and loops in polyps and over 90% in cancer showed reduced signals (Fig. 3a and Extended Data Fig. 5a). Among the annotated CREs, gene coding transcription start sites (TSSs) exhibited an exceptionally high loss rate of stripe strength (Extended Data Fig. 5b). Consistently, the global P–E connectivity was progressively lost in advanced stages, suggesting reduced P–E communications associated with stripe loss (Fig. 3b,c). To test whether these alterations are because of increased chromosomal rearrangements along with stage progression, we applied EagleC^41^ to the mHi-C results, identifying 1–17 structural variants (SVs) in each sample (Extended Data Fig. 5c). The sparsity of the SVs and a comparable number of average events between the mucosa (2.5) and polyp (2.5) stages suggests that they are unlikely a primary driving factor for the genome-wide loss of interactions.

To investigate whether the loss of P–E connectivity was because of changes in the activity of the regulatory elements, we aligned the results with the chromatin accessibility and methylation profiles. In both polyps and adenocarcinoma, alterations in connectivity were poorly correlated with the accessibility changes of both gene promoters and neighboring enhancers (*ρ* ≤ 0.13) (Extended Data Fig. 5d). We also observed a mild progressive loss of accessibility on the promoters but not on the enhancers (Fig. 3c). However, the degree of accessibility loss (−12.3%) was marginal compared to the substantial losses of P–E connectivity (−39.3%) and promoter stripe strength (−41.8%), suggesting underlying factors that specifically contributed to the impairment of distal interaction. On the other hand, hypermethylated and hypomethylated promoters were associated with high and low connectivity losses, respectively (Fig. 3d), consistent with the well-characterized repressive function of DNA methylation^42^. However, for the majority (>80%) of the promoters that were neither hypomethylated nor hypermethylated, we found that demethylated promoters were also associated with a higher rate of connectivity loss compared to methylated ones (Fig. 3d and Extended Data Fig. 5e). Furthermore, demethylated promoters that are hypermethylated in the advanced stages are implicated by their significantly lower initial connectivity in the mucosa samples (Extended Data Fig. 5f). These results together indicate a common factor driving both the global connectivity loss of promoters and the selective hypermethylation of low-connectivity ones rather than hypermethylation as a driving force of the connectivity loss.

Recent studies of clusters of enhancers, also known as superenhancers, suggested that the high valency and number of components in enhancer clusters increased their cooperativity through phase separation^43,44^. Inspired by this observation, we examined whether the stability of the P–E networks was affected by the valency of the networks. Interestingly, we found that a high valency of interacting promoters but not enhancers was associated with a lower rate of connectivity loss from mucosa to adenocarcinoma (Fig. 3e, f). This suggests that cooperative P–P interaction networks are associated with elevated stability during neogenic progression.

### Initial P–E connectome primes subsequent gene dysregulation

**Extended Data Fig. 6:**
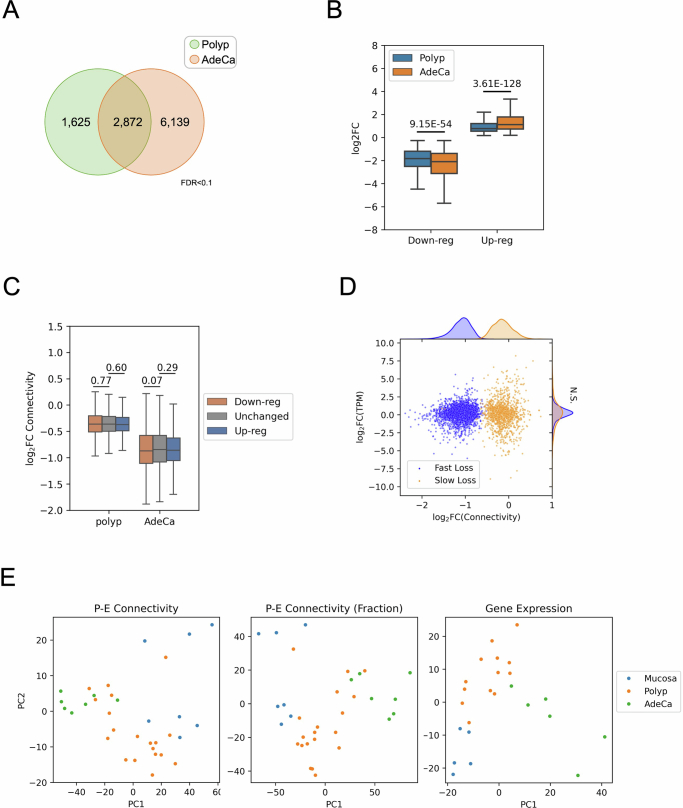
Disconnection between promoter-enhancer (P-E) connectivity and gene expression changes throughout CRC progression. (**a**) Venn diagram illustrating the commonality of genes with significantly modified expression in both polyps and adenocarcinoma (AdeCa). (**b**) Log2 fold changes of gene expression for genes that are consistently up- (N = 1,550) or down-regulated (N = 1,272) across both stages. Statistical significance of the difference in fold changes is assessed using the Wilcoxon signed-rank test. Up-regulated genes: p = 9.15E-54; down-regulated genes: p = 3.61E-128. (**c**) Comparisons of P-E connectivity changes for genes that are up- (N = 1,523/3,212) or down-regulated (N = 1,270/2,061) in polyps/AdeCa, relative to genes with no significant alteration in expression (N = 9,250/6,767). The significance of connectivity changes is evaluated using the Mann–Whitney U test. Between down-reg and unchanged, p = 0.77 and p = 0.07; between up-reg and unchanged, p = 0.60 and p = 0.09 in polyp and AdeCa, respectively. (**d**) Two-dimensional scatter plots and density distributions correlating the changes of top fast-loss (N = 1,000) and slow-loss (N = 1,000) genes in connectivity and gene expression between mucosa and adenocarcinoma. Genes are categorized based on the rate of connectivity loss: fast (blue) and slow (orange), as determined by their feature importance on the first principal component (PC). (**e**) Principal component analysis (PCA) comparing P-E connectivity, scaled P-E connectivity (normalized against the aggregate sum), and gene expression changes during the stages of CRC development. Source data

To elucidate the implications of P–E connectivity on transcriptional outcomes, we analyzed 2,872 genes that exhibited progressive upregulation or downregulation in polyps and adenocarcinomas (Extended Data Fig. 6a,b). We discovered that both upregulated and downregulated genes displayed a similar degree of connectivity loss compared to the genome average (Extended Data Fig. 6c). Consistently, genes associated with increased or decreased connectivity loss did not correlate with upregulation or downregulation (Extended Data Fig. 6d), indicating that the direct impact of P–E connectivity changes on differential gene expression was insignificant. Importantly, however, the first principal component of both the transcriptome and the P–E connectome revealed a consistent trajectory of stage progression, suggesting that the remodeling of the two ‘omes’ during CRC development was closely related, despite their low linear correlation at each individual genes (Extended Data Fig. 6e).

**Fig. 4:**
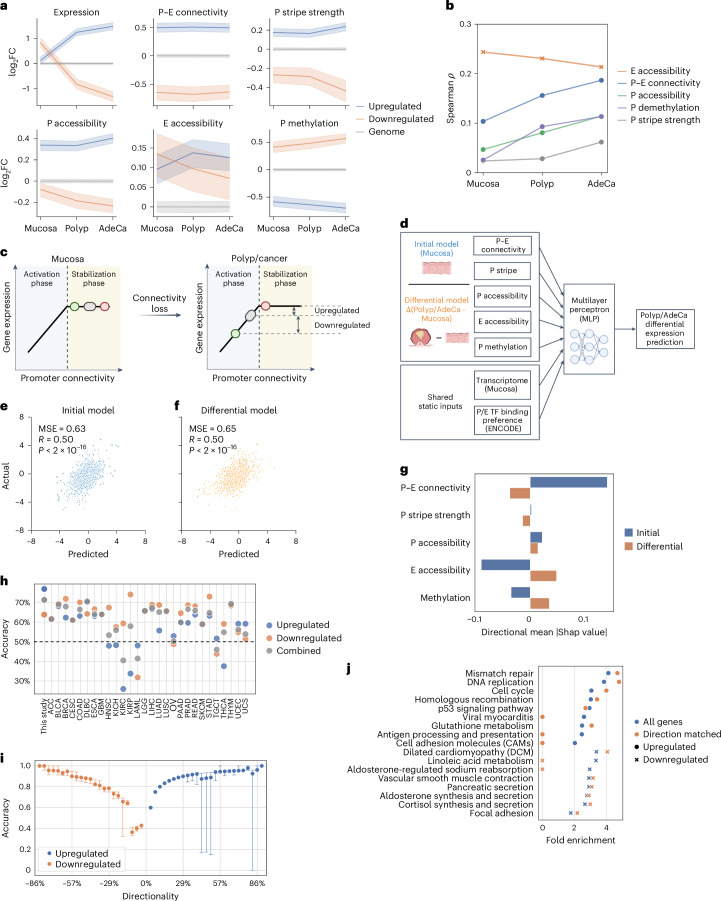
Predictable cancer gene dysregulation by initial P–E connectivity. **a**, Mean relative fold changes of features for genes upregulated (*n* = 1,089) or downregulated (*n* = 944) in both polyps and adenocarcinoma, compared to the genome average at each stage, with confidence intervals shown as shaded areas. **b**, Spearman correlations between transcription levels of active genes (TPM > 0.5) and their structural and epigenetic features at different stages of progression. **c**, Schematics for the two-phase model. In normal conditions, most genes are in the saturated stabilization phase, where increased levels of P–E connectivity stabilize the networks but do not contribute to higher gene expression. In polyp and cancer conditions, genes shift to the activation phase because of global losses of the connectivity, where expression levels are rate-limited by the connectivity levels. Alterations of gene expression during stage progression are, therefore, determined by their initial distance to the activation phase at normal condition. **d**, Diagram illustrating the construction of initial and differential prediction models. **e**,**f**, Predictive accuracy of the initial model for gene expression changes (*r* = 0.50, *P* < 2 × 10^−16^) in polyps (**e**) and the differential model (*r* = 0.50, *P* < 2 × 10^−16^) (**f**) for a test set of genes (*n* = 2,800). **g**, The importance of features and the average direction of association of structural and epigenetic features in the predictive models. **h**, Accuracy of the initial mucosa–polyp model in predicting the direction of significant expression changes in 28 cancer types from TCGA database. **i**, Prediction accuracy for genes (*n* = 13,239) grouped by their directionality scores. Whiskers indicate 1.5× the interquartile range. **j**, Pathway ontology analysis for genes with altered expression in any TCGA cancer type versus those with accurately predicted directional changes by the initial model. Zero values indicate no significant enrichment (FDR > 0.1). Source data

**Extended Data Fig. 7:**
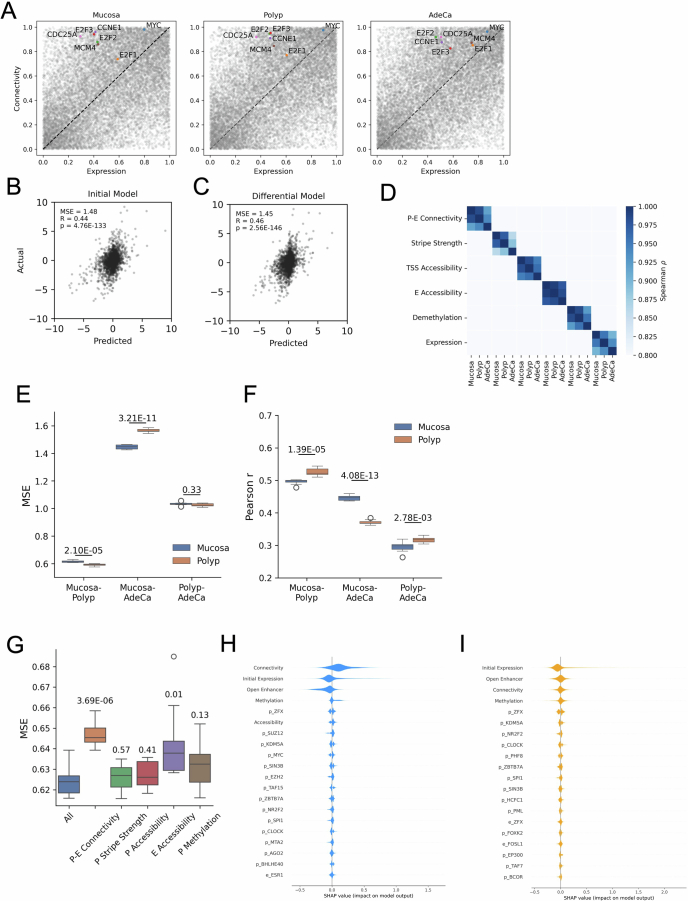
Predictive modeling of gene expression changes based on promoter-enhancer (P-E) connectivity. (**a**) Distributions of ranks for P-E connectivity and corresponding gene expression levels of key oncogenes and proliferation markers across various cancer progression stages. (**b**) Model fit assessment for the predicted changes in gene expression in adenocarcinoma using the ‘initial’ model, which utilizes the baseline P-E connectivity. (**c**) Model fit assessment for the predicted changes in gene expression in adenocarcinoma using the ‘differential’ model, which considers changes in epigenetic landscapes. (**d**) Spearman correlation matrix showing the similarity of each feature between different stages. (**e**) Mean squared error (MSE) and (**f**) Pearson’s r coefficient of the ‘initial’ model for the prediction of gene expression changes in adenocarcinoma compared to the indicated baseline stages. Prediction scores obtained by models trained with mucosa and polyp datasets were compared by using independent t test (N = 10 random initiation states). For mean square error (MSE), p = 2.10E-5, p = 3.21E-11, and p = 0.33; for Pearson’s r, p = 1.39E-5, p = 4.08E-13, and p = 2.78E-3, for mucosa-polyp, mucosa-AdeCa, and polyp-AdeCa prediction models, respectively. (**g**) Distributions of minimal mean square error (MSE) of ‘Initial’ model trained with equal or less than 20 epochs (N = 10 random initiation states) with the removal of indicated features. Significance p values of differential MSE caused by missing features compared to complete model (All) are evaluated by using independent t-test. Respectively, p = 3.69E-6, p = 0.57, p = 0.41, p = 0.01, and p = 0.03 for models removing P-E connectivity, P stripe strength, P accessibility, E accessibility, and P methylation feature. (**h**) The top 20 influential features impacting gene expression predictions in adenocarcinoma, as determined by SHAP (SHapley Additive exPlanations) analysis for the ‘initial’ model. (**i**) The top 20 influential features for the ‘differential’ polyp model, with features named after transcription factors indicating their binding presence at the promoter (p) or enhancer (e) regions, based on the ENCODE database. Source data

Upon investigating the potential nonlinear relationship between P–E connectivity and gene expression, we identified a correlation between differential gene expression and the levels of their connectivity relative to the genome average, as well as to their transcription levels (Fig. 4a). While upregulated and downregulated genes were associated with high and low connectivity, respectively, the connectivity levels were established in unaffected mucosa samples rather than gained or lost in advance stages. As the stage progressed, the gene expression shifted toward higher correlation with the levels of P–E connectivity (Fig. 4b and Extended Data Fig. 7a). Interestingly, a similar trend was also observed between gene expression and other indicators of promoter activity, such as accessibility, stripe activity and demethylation, but not with the accessibility of neighboring enhancers (Fig. 4b). Collectively, these observations suggest a scenario in which impaired P–E connectivity in polyps and adenocarcinomas correlated with an increased dependence of transcription dosage control to the promoter activity.

### A two-phase model predicts dysregulation conserved in cancers

The association of cancer dysregulation with the P–E connectome before cancer development can be explained by a two-phase model. In unaffected mucosa, high P–E connectivity displays functional redundancy, which does not drive high gene expression but rather increases the stability of the P–E networks (stabilization phase). With the loss of connectivity during development of malignancy, the impaired connectivity of the promoters becomes a rate-limiting factor (activation phase), thereby establishing an increased linear correlation with gene expression (Fig. 4c).

A major inference from the two-phase model is that P–E connectivity in baseline conditions primes differential gene expression upon global connectivity loss, highlighting its predictive power for cancer gene dysregulation. To test this hypothesis, we developed an ‘initial’ machine learning model using connectivity and other omics landscapes in mucosa to predict gene expression changes in advanced stages (Fig. 4d). The predicted fold changes exhibited moderate but highly significant correlation for the test gene set in both polyps (*r* = 0.50) and adenocarcinoma (*r* = 0.44) (Fig. 4e and Extended Data Fig. 7b). The initial model performed comparably to a ‘differential’ model, which was trained using the fold change of the epigenetic landscapes during malignancy progression, suggesting that the baseline levels and alterations of the epigenetic landscapes explained a comparable portion of cancer gene dysregulation (Fig. 4f and Extended Data Fig. 7c).

Interestingly, when the baseline of the initial model was changed from mucosa to polyp, which molecularly more closely resembles adenocarcinoma, the prediction accuracy significantly worsened (Extended Data Fig. 7d–f). This suggests that the predictive information in the epigenetic landscape diminishes as oncogenic progression advances. We identified P–E connectivity as the most critical predictive feature in the initial model by sequentially removing each feature, reinforcing its pivotal role in the two-phase model (Extended Data Fig. 7g). The analysis of feature importance revealed distinct predictive factors by the initial and differential models. While the initial model suggested that upregulation was predicted by high P–E connectivity and low enhancer accessibility in unaffected mucosa, the differential model suggested that it was associated with their loss and gain, respectively, in advanced stages (Fig. 4g and Extended Data Fig. 7h,i).

**Extended Data Fig. 8:**
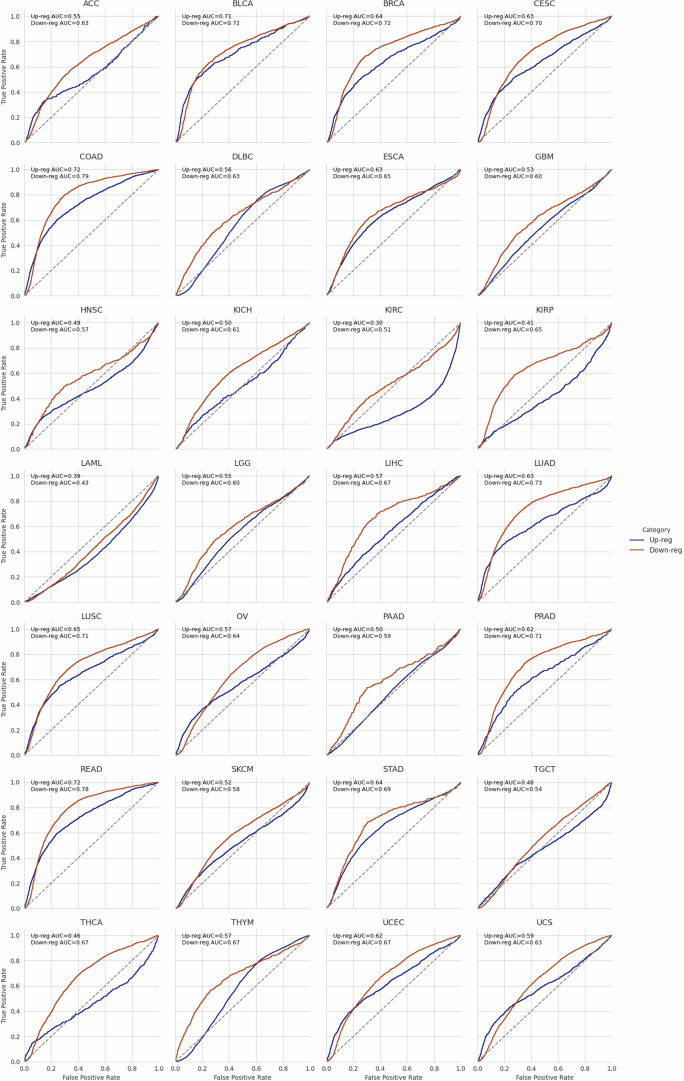
Assessment of predictive accuracy for gene expression changes in various cancer types. ROC curve analysis using gene expression predictions derived from the ‘initial’ polyp model to determine the up- and down-regulation status of genes across different cancer types represented in the TCGA database. Source data

Recent studies on cancer dysregulation have inferred that oncogenic mutations converge on dysregulation of key transcriptional regulators, such as *MYC*^45^ and *E2F* (ref. ^46^), which drive cell proliferation and survival. To test whether the two-phase model is a potential addiction mechanism adopted by cancers to gain proliferative advantages, we applied the initial model to predict gene dysregulation in other cancer types from The Cancer Genome Atlas (TCGA) database^47,48^. We found that the model had generic predictability for significantly dysregulated genes (10/28, area under the curve (AUC) > 0.6; Extended Data Fig. 8) and the directionality of their differential expression (18/28, accuracy > 60%; Fig. 4h). The prediction accuracy further increased toward nearly 100% for genes showing consistent upregulation or downregulation among different cancer types (Fig. 4i). The genes with accurately predicted upregulation were highly enriched in cell cycle and DNA maintenance pathways (Fig. 4j). These results suggest that a substantial portion of conserved cancer gene dysregulation may be explained by the transition of P–E connectivity to the activation phase, including the upregulation of key transcriptional addiction pathways, such as cell proliferation.

### Two-phase model predicts gene-specific intervention sensitivity

**Fig. 5:**
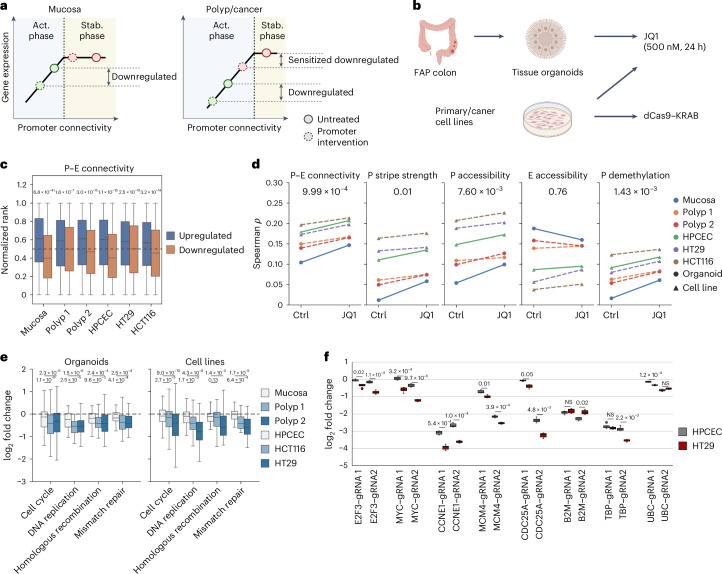
Two-phase model predicts gene- and polyp/cancer-specific sensitivity to inhibitions. **a**, Schematic representing the prediction model. Low-connectivity genes (green dots) are vulnerable to perturbations in the activation phase, independent of overall connectivity levels. Conversely, genes with high connectivity exhibit stage-specific sensitivity to perturbations as they approach the phase transition threshold because of connectivity loss. **b**, Diagram of experimental designs for assessing gene expression sensitivity to various interventions. **c**, Comparisons of distribution of P–E connectivity levels in unaffected mucosa for genes upregulated (*n* = 5,504) or downregulated (*n* = 7,028) after JQ1 treatment of samples. *P* values for significance were determined by the Mann–Whitney *U*-test. Organoid (mucosa), *P* = 6.8 × 10^−41^; organoid (polyp 1), *P* = 1.6 × 10^−7^; organoid (polyp 2), *P* = 3.0 × 10^−16^; HPCEC, *P* = 1.1 × 10^−35^; HT29, *P* = 2.5 × 10^−10^; HCT16, *P* = 3.2 × 10^−14^. **d**, Spearman correlation between structural and epigenetic features in mucosa and gene expression levels in cell lines and organoids before and after JQ1 treatment. *P* values for significance from the Wilcoxon signed-rank test are indicated. P–E connectivity, *P* = 9.99 × 10^−4^; promoter stripe strength, *P* = 0.01; promoter accessibility, *P* = 7.60 × 10^−3^; enhancer accessibility, *P* = 0.76; promoter demethylation, *P* = 1.43 × 10^−3^. **e**, Expression fold change distributions for genes (*n* = 205) in specified pathways following JQ1 treatment. *P* values for sample differential responses from the Wilcoxon signed-rank test are denoted. Respective *P* values for comparisons between mucosa organoid and two polyps: 1.1 × 10^−9^ and 2.3 × 10^−6^ for cell cycle, 2.5 × 10^−5^ and 1.5 × 10^−6^ for DNA replication, 9.6 × 10^−3^ and 2.4 × 10^−4^ for homologous recombination and 4.1 × 10^−4^ and 2.5 × 10^−4^ for mismatch repair. Respective *P* values for comparisons between HPCEC and HT29/HCT116 cell lines: 2.7 × 10^−3^ and 9.0 × 10^−10^ for cell cycle, 1.7 × 10^−3^ and 4.3 × 10^−8^ for DNA replication, 0.13 and 1.4 × 10^−5^ for homologous recombination and 6.4 × 10^−4^ and 1.7 × 10^−5^ for mismatch repair. **f**, Differential gene expression following Cas9–KRAB-mediated repression using two gRNAs in primary human colon epithelial cells (HPCEC) and the HT29 colorectal adenocarcinoma cell line. Measurements were repeated for cells cultured separately (*n* = 8). The significance of differential responses was assessed by a two-sample *t*-test followed by Bonferroni correction. Respective adjusted *P* values for gRNA1 and gRNA2: 0.02 and 1.1 × 10^−4^ for *E2F3*, 3.2 × 10^−4^ and 9.6 × 10^−6^ for *MYC*, 5.4 × 10^−4^ and 1.0 × 10^−4^ for *CCNE1*, 0.01 and 3.9 × 10^−4^ for *MCM4*, 0.05 and 4.8 × 10^−3^ for *CDC25A*, NS and 0.02 for *B2M*, NS and 2.2 × 10^−3^ for *TBP* and 1.2 × 10^−4^ and NS for *UBC*. Source data

In the two-phase model, genes with high P–E connectivity are associated with high stability and are, thus, more transcriptionally resilient to connectivity loss. This model predicts specific transcriptional outcomes driven by connectivity interventions. First, the genome-wide perturbation of the P–E connectome will result in gene-specific expression changes, where low-connectivity genes will be selectively prone to downregulation. Second, high-connectivity genes will be sensitized to perturbations in polyps and cancer because of their shift toward the activation phase along with global connectivity losses (Fig. 5a).

**Extended Data Fig. 9:**
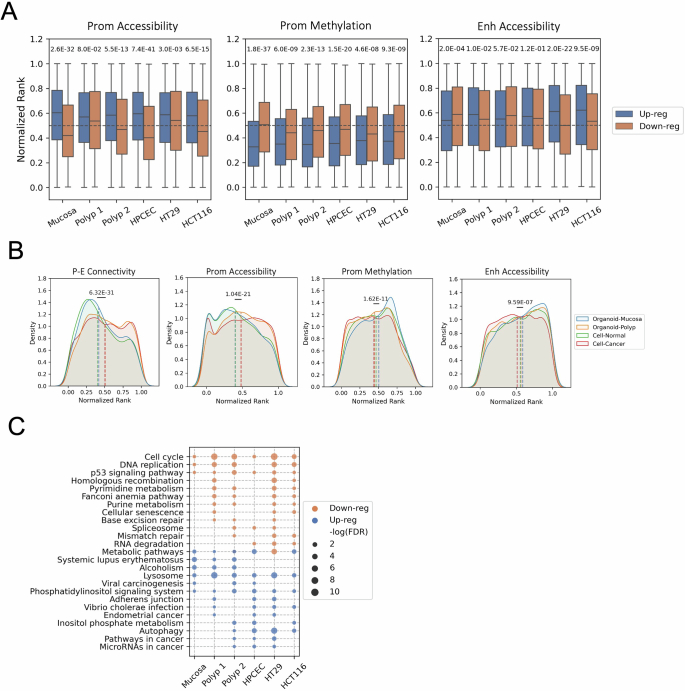
Transcriptomic alterations following JQ1 treatment. (**a**) Distribution of epigenetic feature ranks in unaffected mucosa for genes that are up- (N = 5,504) or down-regulated (N = 7,028) subsequent to JQ1 treatment. P values indicating statistical significance between the two groups are calculated using the Mann–Whitney U test. Respective p values for comparisons of promoter accessibility, promoter methylation, and enhancer accessibility: p = 2.6E-32, p = 1.8E-37, p = 2.0E-4 for organoid (mucosa); p = 0.08, p = 6.0E-9, p = 0.01 for organoid (polyp 1); p = 5.5E-13, p = 2.3E-13, p = 0.057 for organoid (polyp 2); p = 7.4E-41, p = 1.5E-20, p = 0.12 for HPCEC; p = 3.0E-3, p = 4.6E-8, p = 2.0E-22 for HT29; p = 6.5E-15, p = 9.3E-9, p = 9.5E-9 for HCT116. (**b**) Comparative density plots illustrating the differences in feature rank distributions for genes down-regulated in normal tissue (mucosa organoids or primary colon epithelial cells, N = 3,207) versus diseased states (polyp organoids or cancer cell lines, N = 6,007). P values for statistical significance are derived from the Mann-Whitney U test. Individual p values: p = 6.32E-31 for P-E connectivity, p = 1.04E-21 for promoter accessibility, p = 1.62E-11 for promoter methylation, p = 9.59E-7 for enhancer accessibility. (**c**) Pathway analysis based on ontology for genes that are up- or down-regulated in various samples following JQ1 treatment. Source data

To validate these predictions, we first examined the transcriptomic response of colon samples to JQ1 treatment (Fig. 5b). JQ1 is a specific and potent inhibitor of bromodomain and extraterminal domain (BET) family members^49–52^, such as bromodomain-containing protein 4 (BRD4), which has a crucial role in recruiting the mediator complex that bridges P–E interactions^53,54^. In primary and colon cancer cell lines, as well as in organoids derived from mucosa and polyp tissues, genes upregulated or downregulated by JQ1 treatment were consistently associated with high and low initial P–E connectivity, respectively (Fig. 5c). Furthermore, correlations of transcription levels with P–E connectivity and other indicators of promoter activity, but not with the enhancer context, were increased with JQ1 treatment (Fig. 5d and Extended Data Fig. 9a). These results resembled the events during malignancy progression (Fig. 4a,b), suggesting that gene expression alterations induced by JQ1 could be explained by the two-phase model.

By comparing the P–E connectivity distribution of downregulated genes in normal versus polyp and cancer samples, we found a significant increase in the fraction of high-connectivity genes in disease samples (Extended Data Fig. 9b). This result matched the malignancy-specific sensitivity of highly connected genes to perturbations predicted by the two-phase model. Interestingly, genes that were selectively downregulated in polyps and cancer cells were enriched in cell cycle and DNA damage repair pathways (Extended Data Fig. 9c). These pathways responded to JQ1 with a significantly higher fold decrease in disease samples (Fig. 5e), suggesting that, while cell proliferation genes are commonly upregulated during oncogenesis (Fig. 4J), their susceptibility to transcriptional perturbations also increases with impaired P–E connectivity.

**Extended Data Fig. 10:**
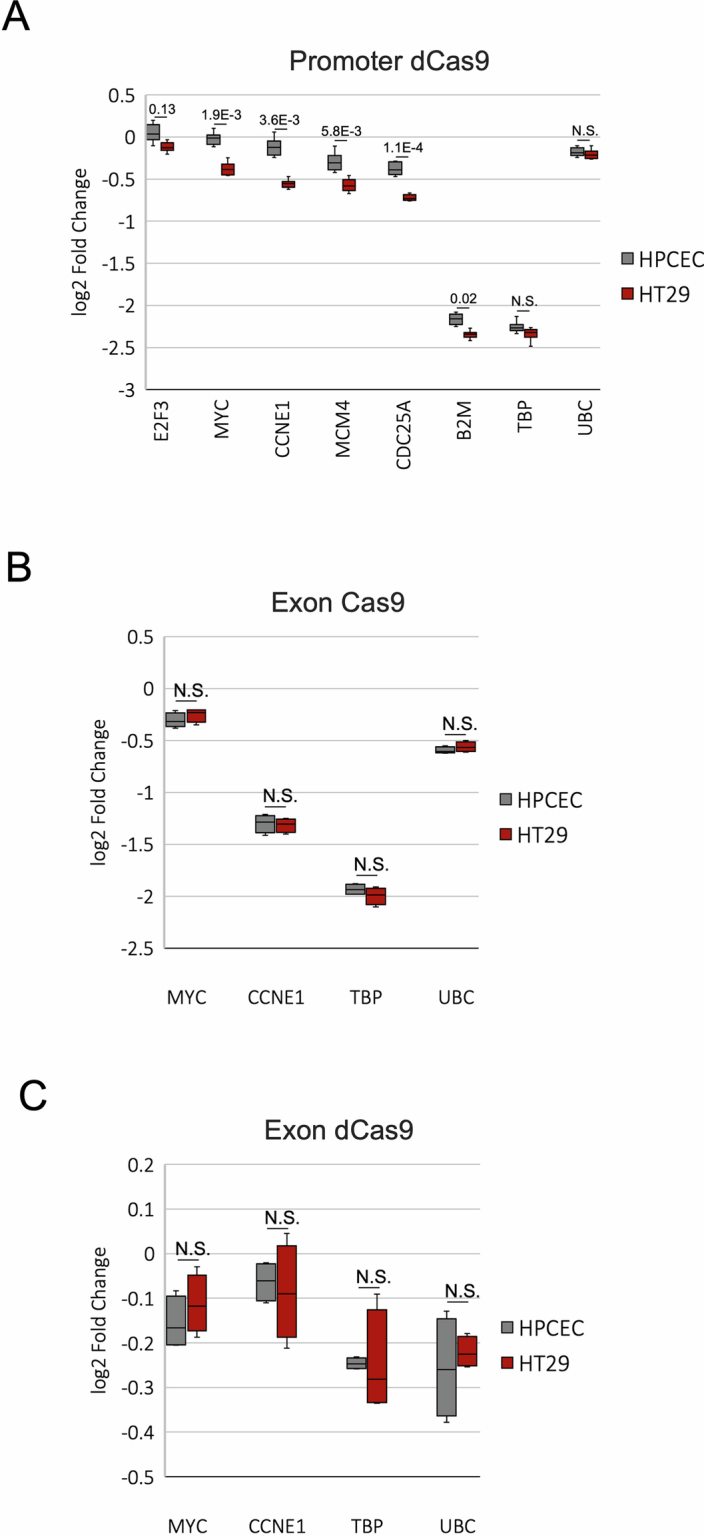
Gene expression changes following Cas9-mediated perturbations in HPCEC and HT29 cells. (**a**) Changes in gene expression after introducing dCas9-gRNA ribonucleoproteins (RNPs) targeting promoters in wild-type cell lines (N = 8). For E2F3, MYC, CCNE1, MCM4, CDC25A, B2M, TBP, UBC, p = 0.13, p = 1.9E-3, p = 3.6E-3, p = 5.8E-3, p = 1.1E-4, p = 0.02, p = 0.56 (N.S.), p = 1 (N.S.), respectively. (**b**) Changes in gene expression after introducing Cas9-gRNA RNPs targeting exons in wild-type cell lines (N = 4). For all comparisons, p = 1 after multiple testing correction. (**c**) Gene expression alterations upon gRNA delivery targeting exons in cell lines stably expressing dCas9-KRAB (N = 4). For all comparisons, p = 1 after multiple testing correction. N numbers indicate replication of measurements in cells cultured separately. Statistical significance of the differential response was assessed using a two-sample t-test followed by Bonferroni correction. Source data

To test whether the cancer-specific perturbation sensitivity can be targeted with a gene-specific strategy, we applied clustered regularly interspaced short palindromic repeats (CRISPR) interference (CRISPRi)^55^ to target the promoters of five proliferation genes (*E2f3a*, *MYC*, *CCNE1*, *MCM4* and *CDC25A*), which were highly connected and correctly predicted to be upregulated in both polyps and adenocarcinoma (Extended Data Fig. 6a and Supplementary Table 2). Comparing transcriptional responses in primary colon epithelial cells (HPCEC) and colon cancer cells (HT29), we found that each of the genes were consistently repressed with a larger effect size in the cancer cell line (Fig. 5f). In contrast, for the three reference genes (*B2M*, *TBP* and *UBC*), neither or only one of the two guide RNAs (gRNAs) targeting each gene showed increased repression fold changes in cancer. These results were reproduced by delivering dCas9–gRNA ribonucleoprotein complexes through electroporation, replacing the lentiviral-delivered Cas9–KRAB (Krüppel-associated box) cassette (Extended Data Fig. 10a). By contrast, gene repression and deletion using the exon-targeting Cas9–dCas9 resulted in similar degrees of downregulation between normal and cancer cell lines (Extended Data Fig. 10b,c), suggesting that the observed difference in repression efficiency was specific to the promoters and not confounded by the delivery efficiency of the system between cell lines. Taken together, consistent with the two-phase model prediction, proliferation genes in cancer showed increased susceptibility to promoter inhibition.

## Discussion

In this study, we provided a comprehensive and integrative analysis of P–E connectivity in conjunction with the transcriptional and epigenetic state of regulatory elements during the early stages of CRC development. Our high-resolution chromatin conformation data, facilitated by multirestriction digestion, revealed a large number (>250,000) of dot interactions and architectural stripes associated with active regulatory elements, such as promoters and enhancers. This represents a pivotal departure from previous descriptions of chromatin architecture that primarily focused on CTCF loop structures and large domain regions^30,56–58^. Our findings indicate that most P–E loops coexisted with stripe formation, suggesting that these interactions are highly dynamic, occurring as either anchor sliding over the intervening chromatin. This challenges the traditional view of stable P–E loops and aligns with the recent proposal of the ‘hub’ model^59,60^, which describes the close vicinity but not tight looping of *cis*-regulatory hubs. Importantly, our study shows that P–E connectivity has an important inference to gene expression dysregulation in cancer, underscoring its fundamental role in gene regulation.

Our results elucidate the distinct roles of P–E connectivity and enhancer activity in CRC progression. During the transformation of polyps and adenocarcinomas, we observed that upregulation of gene expression was often correlated with increased activity of the neighboring enhancer contexts (Fig. 4a,g). Conversely, P–E connectivity diminished for most genes, irrespective of their expression changes (Extended Data Fig. 6c). This intriguing dichotomy underscores the existence of specific regulatory mechanisms governing connectivity that are integral to gene expression and CRC development. The attenuation of P–E connectivity is primarily linked to the reduction in stripe activity on gene promoters (Fig. 3c). However, the precise mechanisms driving the disproportionately high loss rate of connectivity of promoters among CREs remain elusive (Extended Data Fig. 5b). Recent investigations have spotlighted the role of chromatin-binding factors such as polymerase (Pol) II, the Mediator complex and YY1 in sustaining P–E interactions^53,61,62^. Additionally, it has been posited that the cohesin complex retention on promoters is modulated by general transcription activities^2,63^. This suggests that a myriad of transcriptional regulators could influence P–E connectivity, thus potentially leading to therapeutic targets.

We observed an intricate and nonlinear relationship between P–E connectivity and transcription. The similar degree of connectivity loss was associated with both upregulation and downregulation and our analysis revealed that this gene-to-gene variation was correlated with their connectivity levels established in the unaffected mucosa before cancer development. We propose that this nonlinear relationship is a result of a transition in the connectome–transcriptome relationship during CRC development. In the unaffected FAP mucosa, transcription levels are not rate-limited by high P–E connectivity. This redundancy is consistent with previous observations of low correlation between P–E interaction and gene expression^12,13,22,23^. However, our results indicate that the scenario is altered in polyps and cancers, where the loss of connectivity becomes a limiting factor for transcriptional regulation and, thus, correlates with gene expression. Thus, the *cis*-regulatory connectivity has a pivotal role in gene dysregulation associated with cancer progression.

On the basis of the two-phase model, we reasoned that genes with high and low P–E connectivity at baseline condition would be primed for upregulation and downregulation, respectively, upon global connectivity loss or perturbation. This insight was corroborated by the correlation between transcriptional alterations caused by BRD4 inhibition through JQ1 treatment and the initial P–E connectivity levels in unaffected mucosa. Notably, while previous research has reported a comparable number of upregulated and downregulated genes in response to JQ1 treatment, the mechanistic underpinnings of widespread gene upregulation following the inhibition of BRD4, a general transcriptional activator, remained elusive^64,65^. Our study provides a possible explanation, suggesting that the upregulation of genes can be attributed to their promoters’ tolerance to BRD4 inhibition compared to the rest of the genome.

Notably, we identified early established high P–E connectivity in unaffected mucosa tissue as a hallmark of gene upregulation during oncogenic progression. Our pan-cancer analysis suggests that this hallmark is important in multiple cancer types and this finding was particularly pronounced in key transcriptional regulators of proliferation, such as *E2F* and *MYC* (Extended Data Fig. 7a). Previous studies have often described the upregulation of *MYC* and *E2F* as an outcome of genetic mutations or alterations in their upstream regulators^45,66–68^. However, our results suggest an alternative perspective, where the global remodeling of regulatory connectivity has a fundamental role in their frequent upregulation in cancers.

Interestingly, our findings suggesting that P–E connectome remodeling serves as a positive driver in oncogenesis are in contrast with a recent topological study of colon cancer, which proposed a tumor-suppressive effect of large-scale architectural reorganization^56^. This apparent discrepancy likely reflects the distinct influences of macroscopic chromatin structures in previous studies and the microscopic P–E interactions in cancer progression identified in our study. While compartmental remodeling within repressive domains coincided with their hypomethylation and gene repression, we observed a concurrent loss of fine-scale connectivity among active regulatory elements, shifting the global transcriptional balance.

One limitation of our study is its reliance on colorectal tissues from a relatively small cohort of persons with FAP. Previous studies have shown that even seemingly unaffected intestinal mucosa in persons with FAP displays deregulated proliferation compared to tissues from genetically normal individuals^69–71^. Whether this predisposition toward tumorigenic transformation is associated with chromosomal conformational remodeling similar to the changes we observed in subsequent stages of progression remains to be explored. Additionally, while our two-phase model was robust across several cancer types, it did not effectively predict gene dysregulation for certain cancers such as kidney carcinoma and myeloid leukemia (Fig. 4h). This discrepancy may indicate cell-type-specific variations in P–E connectivity, underscoring the need for comparative studies involving these cancers and their respective healthy controls. Future research should expand to more diverse cohorts and cancer origins to fully assess the complex relationship between regulatory connectivity and gene dysregulation proposed by our two-phase model.

In summary, our study offers valuable insights into the complex interplay between 3D genome architecture and gene regulation during the early stages of CRC progression. We provide a unique resource of fine-gauge regulatory architecture that has not been extensively explored in previous cancer chromatin conformation mapping studies. By comprehensively tracing the dynamic changes in P–E connectivity and their impact on gene expression during early CRC development, we identified potential paths for therapeutic interventions. For example, by restoring normal P–E connectivity, it may be possible to interfere with the gene dysregulation events during CRC progression. Further dissection of mechanisms underlying altered *cis*-regulatory connectivity during disease development could identify transcriptional regulators that trigger cancer-specific suppressions of oncogenes, opening up avenues for treatment.

## Methods

### Description of donors

This study was approved by the Stanford Institutional Review Board under protocol no. 47044. Four persons with FAP (one male and three female) were involved in this study (Supplementary Table 1). FAP tissues were collected at the time of partial or full colectomies from participants. Immediately following colectomy, participant-matched non-neoplastic colorectal mucosa, adenomatous polyps and adenocarcinomas were snap-frozen and preserved in liquid nitrogen. One FAP adenocarcinoma (A001-C-007) was embedded in an optimal cutting temperature (OCT) compound before being stored at −80 °C. Sporadic CRCs from six donors were obtained from the Stanford Tissue Bank. Tissues were examined for histopathology to confirm their disease states. Informed consent was obtained from all participants.

### Organoid culture

Tissue samples were collected from participants and processed for organoid generation according to the protocol detailed in Pleguezuelos-Manzano et al.^72^. Briefly, samples were collected on ice in a collection medium (advanced DMEM/F12 supplemented with 10 mM HEPES, 1× Glutamax and 1% penicillin–streptomycin). Tissue samples were washed in collection medium, minced and digested for 30 min at 37 °C in 5 mg ml^−1^ collagenase type II (Sigma-Aldrich). Samples were then filtered using a 100-μm strainer, washed five times in collection medium and plated in Geltrex (ThermoFisher).

Organoids were cultured in a complete medium (advanced DMEM/F12 supplemented with 10 mM HEPES, 1× Glutamax, 1% penicillin–streptomycin, 1× B27 without vitamin A, 10 mM nicotinamide, 1.25 mM *N*-acetylcysteine, 500 nM A83-01 (Tocris), 10 μM SB202190 (Sigma-Aldrich), 100 ng μl^−1^ Noggin (R&D Systems), 1 μg ml^−1^ human recombinant R-spondin (Stemcell), 0.3 nM Wnt-FC (Immunoprecise), 50 ng ml^−1^ EGF (Shenandoah Biotechnology), 2.5 μM CHIR 99021 (Tocris) and 100 μg ml^−1^ Normocin (InvivoGen). Furthermore, 10 μM Y-27632 was added to the medium for the first 3 days after seeding.

For drug experiments, organoids were trypsinized and plated at 30,000 cells per well in 24-well plates. After 5–7 days, organoids were incubated in a complete medium containing 500 nM JQ1 for 24 h. Organoids were then isolated in Cell Recovery Solution (Corning) on ice for 1 h, washed with PBS and centrifuged to retrieve cell pellets. Cell pellets were then processed for RNA extraction.

### Cell lines

HT29 (American Type Culture Collection (ATCC) HTB-38) and HCT116 (ATCC CCL-247) human CRC cell lines were obtained from the ATCC. Cells were maintained in DMEM/F12 (ThermoFisher, 11320033) with 10% FBS and 1% penicillin–streptomycin. Primary human colonic epithelial cells (Cell Biologics, H-6047) were maintained in epithelial cell growth medium (Cell Biologics, H6621) as suggested by the vendor. The identity of all cell lines was authenticated by respective vendors. All cell lines tested negative for *Mycoplasma* and experiments were performed before reaching ten population doublings.

For drug experiments, cells were seeded in six-well plates with 50% confluence. After 1 day, cells were grown in a complete medium containing 500 nM JQ1 for 24 h. Cell pellets were then trypsinized for collection and processed for RNA extraction.

### CRISPR and CRISPRi assays

For stable expression of dCas9–KRAB, pHR-SFFV-KRAB-dCas9-P2A-mCherry (Addgene, 60954) was transfected into HEK293T cells using the Lenti-X packaging single shots (Takara Bio, 631275), following the manufacturer’s protocol. Assembled viral particles were isolated after 72 h of incubation and collected by filtering the culture media through a 0.45-µm filter. Viral titers were determined using the Lenti-X GoStix Plus (Takara Bio, 631280). Viral infections were conducted with a multiplicity of infection of 10, where 2.0 × 10^5^–1.0 × 10^6^ cells were incubated in full culture medium containing the viral particles and 8 µg ml^−1^ polybrene for 48 h. Positive cells were selected on the basis of a high expression of mCherry by fluorescence-activated cell sorting (FACSAria II, BD Bioscience). Cells with stable cassette expression were selected by a second sorting performed 2–3 weeks after the initial.

For delivery of gRNA or Cas9/dCas9–gRNA complex, the synthesized crRNA:tracrRNA duplex (Integrated DNA Technologies (IDT)) was transfected into 1.0 × 10^5^–2.0 × 10^5^ cells using the 4D nucleofector X unit (Lonza) with or without preincubation with equal molar Cas9/dCas9 protein (IDT, 1081058 and 1081066). We followed the protocol provided by IDT for the Lonza nucleofector system. Nucleofection of the cells used the following kits and programs: HPCEC, P3/CM137; HT29, SF/FF137. After nucleofection, cells were seeded in 96-well plates and collected for downstream analysis after 48 h. To obtain statistical robustness, each experiment was repeated with 2–4 trials with two replicates in each trial.

### mHi-C

mHi-C was performed as a derivative of Tri-HiC, a high-resolution modified Hi-C protocol^18,31^, with minor modifications. Initially, 5–10 mg of snap-frozen tissue was placed into a tissueTUBE-TT05 (Covaris, 520071) and cryopulverized using the Covaris CP02 cryoPREP automated dry pulverizer, following the manufacturer’s procedure. The pulverized tissue was then subjected to freeze substitution^73^ by submerging it in 1 ml of −80 °C 0.01% formaldehyde (ThermoFisher, 28906), 97% ethanol and 2% water. Following this, samples were incubated on dry ice for 3 h at a rotor spinning speed of approximately 100 r.p.m. They were then placed in a CoolCell Container (Corning) and transferred to a −20 °C freezer for overnight incubation. On day 2, the container was moved to a 4 °C cold room and spun on a rotor at approximately 100 r.p.m. for 1 h to bring the sample temperature above the freezing point.

Subsequently, the tissue samples were separated from the ethanol solution by centrifuging at 300*g* for 5 min in a 4 °C microcentrifuge. Crosslinking was carried out by incubating the samples with 1 ml of 1% TBS-formaldehyde for 10 min at room temperature. The solution was then quenched by adding 80 μl of 2.5 M glycine and incubated for an additional 5 min. The samples were centrifuged, washed once with 1 ml of TBS (pH 7.5) and resuspended in 250 μl of Hi-C lysis buffer (10 mM Tris-HCl pH 8.0, 10 mM NaCl and 0.2% Igepal CA630) with an additional 50 μl of proteinase inhibitor cocktail (Sigma, P8340). Nuclear extraction was performed on ice by squeezing the samples 15–20 times with 1.5-ml disposable pellet pestles (Fisher Scientific, 12-141-368).

The crude suspension was then centrifuged at 1,500*g* for 5 min at 4 °C, resuspended in 800 μl of Hi-C lysis buffer and passed through a 100-μm strainer (Sysmex). After another centrifugation, the purified nuclei were resuspended in 170 μl of 10 mM Tris-HCl containing 0.5% Triton X-100 (Sigma, 93443). This was followed by incubation at room temperature with rotation for 15 min. Then, 10 μl of 1% SDS, 20 μl of Cutsmart buffer (New England Biolabs (NEB)), 3 μl each of HinP1I (NEB, R0124S), DdeI (NEB, R0175L), CviAII (NEB, R0640L) and FspBI (ThermoFisher, ER1762) and 0.6 μl of MseI (NEB, R0525M) were added to the suspension in the indicated order. The mixture was then incubated at 25 °C and 37 °C for 2 h each with rotation. To halt the restriction digestion, the suspension was incubated in a 62 °C heating block for 20 min, followed by cooling down. End repair was carried out by adding 30 μl of a solution containing 0.5 mM biotin-14-deoxyadenosine triphosphate (Active Motif, 14138), 0.5 mM biotin-14-deoxycytidine triphosphate (AAT Bio, 17019), 0.5 mM deoxythymidine triphosphate, 0.5 mM deoxyguanosine triphosphate and 4 μl of Klenow DNA Pol (NEB, M0210L) to the mixture. This was then incubated for 1 h at 37 °C with rotation. For ligation, a 750-μl solution containing 1× NEB T4 DNA ligase buffer (NEB, B0202), 120 μg of BSA (ThermoFisher, AM2616) and 2,000 U of T4 DNA ligase (NEB, M0202M) was added. The mixture was incubated at room temperature for 90 min, followed by 4 °C overnight and then room temperature for an additional 60 min with rotation.

Reverse crosslinking was performed by centrifuging the mixture at 1,500*g* for 5 min. The supernatant was then replaced with a mixture of 300 μl of 1× T4 ligase buffer, 30 μl of 20 mg ml^−1^ proteinase K (ThermoFisher, 25530049), 30 μl of 10% SDS and 40 μl of 5 M NaCl. This suspension was then incubated at 66 °C for 4 h. The DNA content was purified by phenol–chloroform extraction and resuspended in 20 μl of 10 mM Tris-HCl.

To generate the mHi-C sequencing library, 300 ng of purified DNA was tagmented with 2.5 μl of Tn5 transposase (APExBIO, K1155; discontinued) loaded with equimolar mosaic ends containing Illumina Nextera i5 and i7 extensions, according to the manufacturer’s protocol. The tagmentation was performed in 100 μl of buffer containing 10% DMF, 10 mM Tris-HCl and 150 mM NaCl at 55 °C for 10 min. The product was then column-purified (Zymo D4014) and PCR-amplified for two cycles using the NEBNext master mix (NEB, M0544L) with Illumina Nextera primers and conditions. Biotin enrichment was then performed by adding 20 μl of Dynabeads MyOne Streptavidin C1 (ThermoFisher, 65001) and incubating at room temperature for 30 min with rotation. The magnetic beads were washed three times with 1× wash buffer (10 mM Tris-HCl pH 7.5, 1 mM NaCl and 0.5 mM EDTA) and once with 10 mM Tris-HCl. Final libraries were obtained by amplifying the beads with an additional eight cycles of PCR, followed by purification with solid-phase reversible immobilization (Beckman, B23318) size selection at a 0.5×–1.1× range. The 33 samples were combined into two pools and sequenced using two NovaSeq (Illumina) S4 200-cycle flow cells.

### Real-time PCR and RNA sequencing (RNA-seq)

Total RNA was extracted from approximately 5–10 mg of frozen tissues or approximately 1.0 × 10^5^–1.0 × 10^6^ cells from organoid or cell culture using the Zymo Quick-RNA Miniprep (Zymo R1054), according to the manufacturer’s instructions. After purification, DNA digestion was carried out using the DNA-free DNA removal kit (ThermoFisher, AM1906). For complementary DNA (cDNA) synthesis, up to 1 μg of total RNA was processed using the Superscript IV reverse transcription (RT) system (ThermoFisher, 18091050), with Oligo dT provided in the kit serving as the primer. For RT–PCR, 50 ng of synthesized cDNA was mixed with 10 μl of TaqMan Fast Advanced Master Mix (ThermoFisher, 4444557) and 1× primers and then examined in the QuantStudio 6 Flex system (ThermoFisher). Relative gene expressions were normalized against the internal expression of *GAPDH* using the *ΔΔC*_*t*_ method. Sequencing libraries of mRNA were prepared from 200 ng–1 μg of total RNA using the NEBNext Ultra unstranded preparation kit (NEB, E7775S and E7490S), in accordance with the manufacturer’s protocol. Samples were sequenced on a NovaSeq S1 flow cell for 50-bp paired-end sequencing, resulting in an average of 86.3 million raw paired reads per sample.

### ATAC-seq

ATAC-seq was conducted following the latest ENCODE tissue protocol as described previously^61^. Sequencing was performed on a NovaSeq S1 flow cell using 50-bp paired-end sequencing, resulting in an average of 53.2 million unique fragments mapped for each sample.

### Enzymatic methyl sequencing (EM-seq)

EM-seq was executed as described previously^74^. Libraries were constructed using the NEBNext EM-seq kit (NEB), following the manufacturer’s guidance. Sequencing was carried out using the novel ultrahigh-throughput UG-100 (Ultima Genomics) sequencer.

### mHi-C data processing

Initial processing of mHi-C data was executed using the distiller pipeline (https://github.com/open2c/distiller-nf) with default parameters configured for the SLURM cluster. Deduplicated pair files were then input into Juicer pre^37^ to generate KR-balanced .hic matrices at resolutions of 200 bp, 500 bp, 1 kbp, 2 kbp, 5 kbp, 10 kbp, 20 kbp, 50 kbp, 100 kbp, 250 kbp, 500 kbp and 1 Mbp using a quality score filter of 30. To generate piled-up master matrices for various stages and all samples, pair files were initially merged and sorted using pairtools (https://github.com/open2c/pairtools).

Stripe calling was carried out as previously described^18^, with minor modifications to the parameters. We noted an enrichment of mappable reads at open chromatin regions, raising the possibility of false stripe signal detection by measuring raw read count over-representation. To address this, the stripe-calling algorithm normalized long-range contacts against read mappability at each locus, evaluated by distal interactivity-independent self-ligation events. Specifically, long-range (>1.5 kb) and short-range (<1 kb) mapped read pairs (+ or − orientation) were separated into two .bam files using awk and SAMtools. BEDTools was then used to map these reads to two binning bed tracks: a local one with a 2-kb window and a background one with a 50-kb window, both featuring a 100-bp sliding size. Assuming that the over-representation sourced from mappability was proportional between long-range and short-range contracts, the expected count number for each bin was calculated as (long_bg_/short_bg_) × short_local_. Using MACS2 ‘bdgcmp -m qpois’, the local long-range read count for each bin was examined for statistical significance of enrichment against the expected number. The log fold change signal (stripe strength) was then calculated with the same formula by inputting the actual and expected values into MACS2 ‘bdgcmp -m logFE’. To prevent NaN (not a number) errors, a pseudo-count of 1 was added.

After the determination of stripe *q* values, each 100-bp bin was counted for the number of samples demonstrating significance (false discovery rate (FDR) < 0.01). Bins with at least three sample hits were deemed significant. These bins were then merged and only windows with a minimum size of 500 bp were included as final stripe anchors. Stripe peaks overlapping with ENCODE blacklist regions were removed. To mitigate gender variations among participants, only autosomal chromosomes were included for downstream analyses.

Loop calling was performed using the HiCCUPS algorithm from Juicer tools^37^ with the following parameters: ‘-r 500,1000,2000,5000,10000 -f 0.1 -p 4,2,2,2,2 -i 20,10,10,6,6 -t 0.1,1.25,1.75,2 -d 2000,2000,4000,10000,20000’. Given that library complexity significantly affects loop calling power, the analysis was not performed for each individual sample. Instead, it was executed on pooled libraries of (1) all samples; (2) all mucosa; (3) all polyps; and (4) all adenocarcinomas. Postprocessed loop pixels from all profiles at various resolutions were subsequently merged in the order of high resolution to low resolution from combined, mucosa, polyp and adenocarcinoma libraries. A loop with lower priority was filtered if both anchors overlapped with a higher-priority loop. This master loop list was then applied to each sample to execute individual loop quantification. Loop strengths were calculated by dividing read counts in the identified loops by the expected count from the donut background and log-transforming the results. A pseudo-count of 1 was added as necessary to prevent NaN errors. For stage-specific counting, loops with average loop strengths greater than 1.2-fold in samples of the specified stage were regarded as positive.

For annotations for stripe and loop anchors, features were mapped against the Ensembl regulatory build^36^ and TSS from Gencode using BEDTools. If an anchor overlapped with multiple features, the primary annotation adhered to the following order: promoter or TSS (within −1.5 kb to +0.5 kb of any Gencode transcript), active enhancers (defined by H3K27Ac marks in the Ensembl regulatory build), CTCF-binding sites and open chromatin.

For aggregation analysis of loops, aggregated peak analysis (APA) from Juicer was conducted with the parameters ‘-r 200 -u -n -0 -w 500 -k KR -q 20’. The enrichment score was computed as the average intensity of the 10 × 10 center pixels (2 kb) against the mean of the 100 × 100 pixels from the bottom left. For the aggregation of stripes, the same function was executed with the parameters ‘-r 200 -u -n -0 -w 250 -k KR -q 20’. Given the phenomenal distance-dependent interaction decay at the vicinity of stripes, interaction intensities at specific distances (that is, matrix diagonals) were normalized against the average intensity of the distance. The fold enrichment of the aggregated stripes was then calculated by averaging the normalized values in the center ten pixels. For visualizations of loop and stripe APA, matrices were log-transformed before being plotted onto heat maps.

For SV calling, contact matrices in .mcool format were generated by using the distiller pipeline, using default parameters as described above. These matrices were analyzed using the PredictSV function in the EagleC package^41^ with the following parameters: ‘--balance-type ICE --output-format full --prob-cutoff-5k 0.8 --prob-cutoff-10k 0.8 --prob-cutoff-50k 0.9999’. Identified SVs were visually confirmed on the Hi-C heat maps.

### ATAC-seq processing

ATAC-seq results were processed using the ENCODE-DCC ATAC-seq pipeline (https://github.com/ENCODE-DCC/atac-seq-pipeline) with default settings. To generate the integrated peak list from all samples, a 100-bp binning track was created and mapped with the pseudo-replicated peak regions from each sample using BEDTools. Bins with at least three hits were deemed valid and merged; intervals with a minimum size of 300 bp were included as final peak sites. Peak fold enrichments of samples were then obtained from pipeline-derived fold change bigWig tracks.

### Analysis of P–E connectivity

To elucidate the connectivity between promoters and their neighboring regulatory context, P–E pairs were determined for all ATAC-seq peaks within a 200-kb distance from target promoters. Interaction zones were defined as being within −1.5 kb to +0.5 kb near the TSS for promoters and within 500 bp of ATAC-seq peaks for distal enhancers. Contact frequencies between promoter and each target enhancer were calculated using Juicer Straw^37^ to extract read counts at 1,000-bp resolution. These raw contacts were normalized against the read mappability of the promoter, which was defined as the KR-normalized contact frequency or, in the case where balanced matrix was not available, the ratio of short-range self-ligation contact (<1.0 kb, + or − orientation) reads per kilobase per million mapped reads at the TSS region versus that for the 50-kb neighborhood. Normalization for coverage between samples was achieved by dividing frequencies of each contact by the long-range contact (with a minimum 1.5-kb distance threshold) densities in the 5–50-kb background donut region, in the unit of contacts per 1 × 1 kb square. The aggregated normalized contacts of the gene with all distal regulatory elements represented the total P–E connectivity of that gene.

The accessibility of interacting regulatory element peaks, referred to as ‘enhancer accessibility’ in the text, was calculated as the log sum of the fold enrichment signals of all ATAC-seq peaks that were involved in calculating the P–E connectivity. This value was extracted from the processed ATAC fold change bigWig tracks using pyBigWig, which represented the overall activity of the regulatory context for each gene, and was used for comparisons with P–E connectivity levels. For promoter accessibility, the top two quantiles of mean fold change of the ATAC-seq signals in the defined TSS region were retrieved using pyBigWig and log-transformed. For downstream analyses, genes showing nonpositive values for P–E connectivity, promoter accessibility or promoter stripe enrichment in all three stages were removed. For the remaining genes, missing or negative values were replaced with zero.

### RNA-seq processing

RNA-seq results were processed using T. Bencomo’s pipeline (https://github.com/tjbencomo/bulk-rnaseq), which uses Salmon for quantifying transcript levels and DESeq2 for identifying differential genes (FDR < 0.1, fold change > 1.3). Transcription levels (TPM) of genes were obtained by summing the transcript-based TPM from the Salmon output (.rf).

### DNA methylation processing

A total of 21,175,510 CpG sites with measurable methylation ratios were identified across all samples. The methylation degree of features, including mHi-C hotspots, ATAC peaks and gene promoters, was calculated by averaging the methylation percentage for all valid CpG sites within the feature. Regions with average methylation < 25% were classified as demethylated, while those > 40% were considered methylated. Regions with average methylation between 25% and 40% were classified as intermediately methylated and excluded from the methyl versus demethyl analyses. For methylation changes between two stages, regions demonstrating a >15% difference with <0.1 FDR were classified as significantly hypomethylated or hypermethylated on the basis of the direction of change. For correlation analyses with other features, the degree of demethylation (100% minus methylation percentage, annotated as ‘demethylation’) was often used to maintain a positive correlation between methylation degree and regulatory activity.

### Mappability analysis

The mappability of mHi-C, in situ Hi-C and MNase-digested Intact Hi-C at annotated regulatory regions was visualized by retrieving their read coverage from a .bw file that compiled only short-range self-ligation contacts, defined as those with an interaction distance of less than 1.5 kb and a + or − orientation. To compare the distal interaction signal against mappability, long-range interactions, defined as those with an interaction distance greater than 2.0 kb, were extracted and aggregated similarly. The fold enrichment of interaction was then calculated as the ratio of long-range to short-range interactions at 100-bp resolution. Note that this ratio is equivalent to the ‘raw’ stripe strength before normalization against the average enrichment of the local background.

### Principal component analysis (PCA)

PCA of mHi-C and RNA-seq was performed using the Python sklearn.decomposition.PCA package. For P–E connectivity, analysis was conducted using either untransformed or scaled-by-sample matrices.

### Motif enrichment analysis

For each feature (P–E connectivity, enhancer accessibility, TSS accessibility, TSS stripe strength, TSS methylation and gene expression), sequences of promoter regions for genes with the top 10% feature strength in mucosa were extracted using BEDTools. Motif enrichment was then calculated using the AME tool in the MEME suite^75^ with the JASPAR 2022 vertebrate motif database serving as the reference. The −log_10_(*P* values) for significantly enriched motifs for any of the features were included for hierarchical clustering.

### Gene ontology

Enrichment analysis of significantly upregulated or downregulated genes in pathways was performed and visualized using the web-based gene set analysis toolkit^76^. The method of over-representation was selected to test enrichments in the Kyoto Encyclopedia of Genes and Genomes pathway against the protein-coding genome. Analysis was performed using default parameters.

### Machine learning

For the initial and differential models, structural and epigenetic feature values in unaffected mucosa or their fold changes in polyps or adenocarcinoma were compiled for each promoter. For fold-change calculation, a pseudo-count of 1 was added to connectivity and methylation to avoid zero values. These features were further compiled with the expression levels of the genes in mucosa, the binary binding status of all TFs in the ENCODE chromatin immunoprecipitation with sequencing (ChIP-seq) database at their promoters and the sum of the binary binding status for the TFs in their distal enhancer contexts.

The raw value and their rank transform were combined, resulting in a total of 1,374 features for each gene. To train the models, 2,800 randomly selected genes were excluded as the test dataset and the rest were fit to the differential gene expression changes in polyps and adenocarcinoma using a sequential model with three intermediate layers and one dense output. Each layer included 2,048, 512 and 128 neurons in order and was filtered with a 20% dropout rate. Models were trained for up to 50 epochs, with the final model represented by the iteration that showed the lowest mean squared error for the test dataset. The training and evaluation of the models were performed using the Tensorflow Keras module in Python.

For evaluation of feature importance in the models, the Shapley additive explanations (SHAP)^77^ package was used for analysis with default parameters. Average feature importance was calculated as the absolute mean of the importance across all genes. Overall directionality was represented by the numerical mean of the importance.

### TCGA gene expression analysis

A list of differentially expressed genes and their fold changes were obtained from the GEPIA database^47^. The prediction of differential expression was performed using receiver operating characteristic (ROC) analysis in the Python sklearn package, where predicted differential fold changes from the initial model were used as thresholds for the upregulated and downregulated genes. For prediction of the directionality, genes were scored by their consistency of dysregulation, averaging their alterations (−1 for downregulation, 0 for unchanged and +1 for upregulation). The correlation between the directionality score and the prediction accuracy was then evaluated.

### Statistics and reproducibility

All statistical analyses were performed using Python, R or Excel. Unless specified in the figure legend, statistical significance was calculated using the Mann–Whitney *U*-test or unpaired *t*-test, assuming a normal distribution of samples, between two experimental conditions. Raw *P* values < 0.05 or adjusted FDR values < 0.1 were considered statistically significant, as indicated in each figure legend. All statistical tests were two-tailed. The exact *P* values are reported in each figure, except where *P* was equal to 1 (not significant (NS)) or below a low-value threshold (2 × 10^−16^).

Sample sizes were predetermined to have a minimal power of 0.8 for a 1.5-fold effect size with a coefficient of variance less than or equal to 0.3. The exact sample sizes are indicated in the figure legends. No data points or outliers were excluded from the experiment. Randomization and blinding of samples were not possible for this study. However, all experiments involving case–control comparisons were performed in the same batch and treated identically without prior designation. In bar plots, values and error bars indicate the sample mean and s.e.m. unless specified otherwise in the figure legend. In box–whisker plots, the center indicates the median value and the bounds of the box are defined by the first and third quartiles. Whiskers are drawn within the 1.5 interquartile range.

### Reporting summary

Further information on research design is available in the Nature Portfolio Reporting Summary linked to this article.

## Supplementary information

Reporting Summary

Supplementary Tables Supplementary Table S1: Metadata of FAP and sporadic CRC donors involved in this study. Supplementary Table 2: List of gRNA sequences and Taqman gene expression primers used for CRISPR and CRISPRi intervention assays. Supplementary Table 3: List of sample identifiers used for this study on the HTAN and GEO portals.

## Source data

Source Data Fig. 1 Statistical source data.

Source Data Fig. 2 Statistical source data.

Source Data Fig. 3 Statistical source data.

Source Data Fig. 4 Statistical source data.

Source Data Fig. 5 Statistical source data.

Source Data Extended Data Fig. 1 Statistical source data.

Source Data Extended Data Fig. 3 Statistical source data.

Source Data Extended Data Fig. 4 Statistical source data.

Source Data Extended Data Fig. 5 Statistical source data.

Source Data Extended Data Fig. 6 Statistical source data.

Source Data Extended Data Fig. 7 Statistical source data.

Source Data Extended Data Fig. 8 Statistical source data.

Source Data Extended Data Fig. 9 Statistical source data.

Source Data Extended Data Fig. 10 Statistical source data.

## Data Availability

Raw and processed sequencing data for ATAC-seq, RNA-seq, EM-seq and mHi-C that support the findings of this study were deposited to the National Center for Biotechnology Information Gene Expression Omnibus (GEO) under accession number GSE207954, the NIH Database of Genotypes and Phenotypes (phs002371) and the NCI HTAN portal (RRID: SCR_023364) (Supplementary Table 3). The human cancer data panel was derived from TCGA Research Network (http://cancergenome.nih.gov/). The dataset derived from this resource that supports the findings of this study is available from the GEPIA database (http://gepia2.cancer-pku.cn/). An unmasked hg38 genome (GCA_000001405.15, UCSC Genome Browser) was used as the reference for all analyses. The regulatory build for the sigmoid colon (version 20210107) was obtained from Ensembl (http://www.ensembl.org) to facilitate regulatory annotations. Gencode v38 was used for RNA-seq alignment and defining the positions of TSSs. The ENCODE blacklist (https://github.com/Boyle-Lab/Blacklist) was used to exclude problematic regions of the genome from analyses. ENCODE in situ Hi-C (ENCSR123UVP) and MNase-digested Intact Hi-C (ENCSR477GZK) datasets for the HCT116 cell line were used for comparison with our mHi-C data. Roadmap histone ChIP-seq tracks for colonic mucosa (GSM1112779, GSM916043, GSM916046 and GSM916045) and ENCODE CTCF (ENCSR833FWC), Pol II (ENCSR322JEO), RAD21 (ENCSR956UIS) and SMC3 (ENCSR149SKU) ChIP-seq were used for CRE visualization. Locations of CpG islands were downloaded from the UCSC Genome Browser. All other data supporting the findings of this study are available from the corresponding author upon reasonable request. Source data are provided with this paper.

## Code availability

Custom code used to analyze data in this study is available from GitHub (https://github.com/kimagure/mHi-C_codes). All other code supporting the findings of this study are available in the Methods or are available from the corresponding author upon reasonable request.
