# Author Correction: Global loss of promoter–enhancer connectivity and rebalancing of gene expression during early colorectal cancer carcinogenesis

**DOI:** 10.1038/s43018-025-00915-4

**Published:** 2025-01-26

**Authors:** Yizhou Zhu, Hayan Lee, Shannon White, Annika K. Weimer, Emma Monte, Aaron Horning, Stephanie A. Nevins, Edward D. Esplin, Kristina Paul, Gat Krieger, Zohar Shipony, Roxanne Chiu, Rozelle Laquindanum, Thomas V. Karathanos, Melissa W. Y. Chua, Meredith Mills, Uri Ladabaum, Teri Longacre, Jeanne Shen, Ariel Jaimovich, Doron Lipson, Anshul Kundaje, William J. Greenleaf, Christina Curtis, James M. Ford, Michael P. Snyder

**Affiliations:** 1 Department of Genetics, Stanford School of Medicine, Stanford, CA USA; 2 Ultima Genomics, Newark, CA USA; 3 Department of Medicine, Stanford School of Medicine, Stanford, CA USA; 4 Department of Pathology, Stanford School of Medicine, Stanford, CA USA; 5 Department of Computer Science, Stanford University, Stanford, CA USA; 6 Chan Zuckerberg Biohub, San Francisco, CA USA

**Keywords:** Chromatin structure, Cancer genomics, Genome-wide analysis of gene expression, Dynamic networks, Cancer

Correction to: *Nature Cancer* 10.1038/s43018-024-00823-z, published online 30 October 2024.

In the version of the article initially published, a technique described as “intact Micro-C” should have been called “MNase-digested Intact Hi-C” and has now been corrected throughout the article.

