# Supplementary material for: Global loss of promoter–enhancer connectivity and rebalancing of gene expression during early colorectal cancer carcinogenesis
Source: Nat Cancer. 2024 Oct 30;5(11):1697–712. doi: 10.1038/s43018-024-00823-z (PMC11584406; doi:10.1038/s43018-024-00823-z)
Supplement: Supplementary file 1 — Reporting Summary [file 43018_2024_823_MOESM1_ESM.pdf]

nature portfolio

Corresponding author(s): Michael P. Snyder

Last updated by author(s): 07/26/2024

Reporting Summary

Nature Portfolio wishes to improve the reproducibility of the work that we publish. This form provides structure for consistency and transparency in reporting. For further information on Nature Portfolio policies, see our Editorial Policies and the Editorial Policy Checklist.

Statistics

For all statistical analyses, confirm that the following items are present in the figure legend, table legend, main text, or Methods section.

n/a	Confirmed	
☐	☒	The exact sample size (*n*) for each experimental group/condition, given as a discrete number and unit of measurement
☐	☒	A statement on whether measurements were taken from distinct samples or whether the same sample was measured repeatedly
☐	☒	The statistical test(s) used AND whether they are one- or two-sided<br>*Only common tests should be described solely by name; describe more complex techniques in the Methods section.*
☒	☐	A description of all covariates tested
☐	☒	A description of any assumptions or corrections, such as tests of normality and adjustment for multiple comparisons
☐	☒	A full description of the statistical parameters including central tendency (e.g. means) or other basic estimates (e.g. regression coefficient) AND variation (e.g. standard deviation) or associated estimates of uncertainty (e.g. confidence intervals)
☐	☒	For null hypothesis testing, the test statistic (e.g. *F*, *t*, *r*) with confidence intervals, effect sizes, degrees of freedom and *P* value noted<br>*Give P values as exact values whenever suitable.*
☒	☐	For Bayesian analysis, information on the choice of priors and Markov chain Monte Carlo settings
☒	☐	For hierarchical and complex designs, identification of the appropriate level for tests and full reporting of outcomes
☐	☒	Estimates of effect sizes (e.g. Cohen's *d*, Pearson's *r*), indicating how they were calculated

Our web collection on statistics for biologists contains articles on many of the points above.

Software and code

Policy information about availability of computer code

Data collection	Sequencing results obtained from the Illumina Novaseq 6000 platform were demultiplexed by bcl2fastq (v2.20)
Data analysis	distiller-nf (0.3.3) pairtools (0.3.0) juicer_tools/HiCCUPS (2.13.06) samtools (1.15.1) bedtools (2.30.0) macs2 (2.2.7.1) straw (0.1.0) pyBigWig (0.3.18) salmon (1.4.0) DESeq2 (1.36.0) WebGestalt (2019) EagleC (v0.1.9) hicstraw (1.3.1) tensorflow (2.11.0) shap (0.45.1) sklearn (1.4.2) ENCODE DCC ATAC-seq pipeline (https://github.com/ENCODE-DCC/atac-seq-pipeline) RNA-seq pipeline (https://github.com/tjbencomo/rna-pipeline) MEME (5.5.4) custom (https://github.com/kimagure/mHi-C_codes)

For manuscripts utilizing custom algorithms or software that are central to the research but not yet described in published literature, software must be made available to editors and reviewers. We strongly encourage code deposition in a community repository (e.g. GitHub). See the Nature Portfolio guidelines for submitting code & software for further information.

Data

Policy information about availability of data

All manuscripts must include a data availability statement. This statement should provide the following information, where applicable:

- Accession codes, unique identifiers, or web links for publicly available datasets
- A description of any restrictions on data availability
- For clinical datasets or third party data, please ensure that the statement adheres to our policy

Raw and processed sequencing data for ATAC-seq, RNA-seq, EM-seq, and mHi-C data that support the findings of this study have been deposited in the NCBI Gene

Expression Omnibus (GEO), accession number GSE207954, dbGAP (phs002371), and on the NCI Human Tumor Atlas Network portal (RRID: SCR_023364) (Table S3). The human cancer data panel was derived from the TCGA Research Network: http://cancergenome.nih.gov/. The dataset derived from this resource that supports the findings of this study is available in the GEPIA database (http://gepia2.cancer-pku.cn/). An unmasked hg38 genome (GCA_000001405.15, UCSC Genome Browser) was utilized as the reference for all analyses. The regulatory build for the sigmoid colon (version 20210107) was obtained from Ensembl (http://www.ensembl.org) to facilitate regulatory annotations. Gencode v38 was employed for RNA-seq alignment and defining the positions of transcription start sites (TSS). The ENCODE blacklist (https://github.com/Boyle-Lab/Blacklist) was utilized to exclude problematic regions of the genome from analyses. ENCODE in situ Hi-C (ENCSR123UVP) and intact micro-C (ENCSR477GZK) datasets for the HCT116 cell line were used for comparison with our mHi-C data. Roadmap histone ChIP-seq tracks for colonic mucosa (GSM1112779, GSM916043, GSM916046, GSM916045) and ENCODE CTCF (ENCSR833FWC), Pol II (ENCSR322JEO), RAD21 (ENCSR956UIS), SMC3 (ENCSR149SKU) ChIP-seqs were employed for CRE visualization. Locations of CpG islands were downloaded from the UCSC Genome Browser. Source data for all figures, including the CRISPR intervention experiment results, have been provided as Source Data files. All other data supporting the findings of this study are available from the corresponding author upon reasonable request.

Research involving human participants, their data, or biological material

Policy information about studies with human participants or human data. See also policy information about sex, gender (identity/presentation), and sexual orientation and race, ethnicity and racism.

Reporting on sex and gender	The study included 4 male, 4 female patients, and two patients with unconfirmed gender. Information of sex and gender is collected but not considered in this study since the disease phenotype of FAP is not known to be gender-specific. For all analyses of chromosomal features in this study, only autosomal chromosomes were considered.
Reporting on race, ethnicity, or other socially relevant groupings	Among four FAP patients, two were White, and two were Hispanic/Latino. Please see the supplemental table for additional information. For sporadic CRC patients, race and ethnicity info was no collected.
Population characteristics	Patients in this study include patients with FAP seen at Stanford healthcare and patients with CRC with samples deposited in the Stanford Tumor bank. Ages of patients included ranged from 20–79.
Recruitment	For the FAP patients, given that this is a relatively rare condition, no criteria other than FAP were required for recruitment. Eligible patients undergoing colonoscopy, pouchoscopy, or colectomy were identified in the Stanford Cancer Genetics clinic, the Stanford Gastroenterology and Hepatology service, or the Stanford Adult and Pediatric Surgery service. Eligible patients were notified of their eligibility to participate in research. Over the phone, the description, risks, benefits, and alternatives of participating in the research study were described and a copy of the full consent form was sent to them via email. On they day of the procedure, they met with a clinical research coordinator to answer questions and sign the consent. This recruitment strategy only includes patient's seen at Stanford healthcare, which is one potential source of selection bias. A subset of CRC samples were obtained from the Stanford tumor bank, with no attempt to exclude patients on the basis of age, gender, or sex.
Ethics oversight	The study was approved by the Stanford IRB (protocol #47044) and informed consent was obtained from all patients.

Note that full information on the approval of the study protocol must also be provided in the manuscript.

Field-specific reporting

Please select the one below that is the best fit for your research. If you are not sure, read the appropriate sections before making your selection.

☒ Life sciences ☐ Behavioural & social sciences ☐ Ecological, evolutionary & environmental sciences

For a reference copy of the document with all sections, see nature.com/documents/nr-reporting-summary-flat.pdf

Life sciences study design

All studies must disclose on these points even when the disclosure is negative.

Sample size	Sample size was determined to achieve 80% power with 5% error rate for detecting 0.5 fold change difference between groups with 0.3 coefficient of variation within each group.
Data exclusions	No data was excluded from the analyses.
Replication	The study was performed in two trials each with distinct samples from two different FAP patient groups. All major findings stated in the study were consistent between the sub-experiments that were together reported.
Randomization	Randomization is not required since the design of the study is supervised learning of differential features between cancer development stages.
Blinding	The sample collection and analyses were unblinded due to necessity for their pathological labeling and optimized design to achieve maximum power of study. This should not impact the analyses since all computations were applied equally to all samples. No analytical procedure was taken for any specific group.

Reporting for specific materials, systems and methods

We require information from authors about some types of materials, experimental systems and methods used in many studies. Here, indicate whether each material, system or method listed is relevant to your study. If you are not sure if a list item applies to your research, read the appropriate section before selecting a response.

Materials & experimental systems

n/a	Involved in the study
☒	☐ Antibodies
☐	☒ Eukaryotic cell lines
☒	☐ Palaeontology and archaeology
☒	☐ Animals and other organisms
☒	☐ Clinical data
☒	☐ Dual use research of concern
☒	☐ Plants

Methods

n/a	Involved in the study
☒	☐ ChIP-seq
☒	☐ Flow cytometry
☒	☐ MRI-based neuroimaging

Eukaryotic cell lines

Policy information about cell lines and Sex and Gender in Research

Cell line source(s)	HT-29 (ATCC HTB-38) and HCT-116 (ATCC CCL-247) human colorectal cancer cell lines were obtained from ATCC. Primary human colonic epithelial cells were obtained from Cell Biologics (Cell Biologics H-6047).
Authentication	Authentication was performed by the suppliers using procedures described in corresponding documentations. All cell experiments were performed before reaching 10 population doublings to minimize any potential phenotypic changes of cells.
Mycoplasma contamination	All cell lines were tested negative for mycoplasma contamination.
Commonly misidentified lines (See ICLAC register)	No commonly misidentified lines were used.

Plants

Seed stocks	*Report on the source of all seed stocks or other plant material used. If applicable, state the seed stock centre and catalogue number. If plant specimens were collected from the field, describe the collection location, date and sampling procedures.*
Novel plant genotypes	*Describe the methods by which all novel plant genotypes were produced. This includes those generated by transgenic approaches, gene editing, chemical/radiation-based mutagenesis and hybridization. For transgenic lines, describe the transformation method, the number of independent lines analyzed and the generation upon which experiments were performed. For gene-edited lines, describe the editor used, the endogenous sequence targeted for editing, the targeting guide RNA sequence (if applicable) and how the editor was applied.*
Authentication	*Describe any authentication procedures for each seed stock used or novel genotype generated. Describe any experiments used to assess the effect of a mutation and, where applicable, how potential secondary effects (e.g. second site T-DNA insertions, mosiacism, off-target gene editing) were examined.*
